# The association between active tobacco use during pregnancy and growth outcomes of children under five years of age: a systematic review and meta-analysis

**DOI:** 10.1186/s12889-018-6137-7

**Published:** 2018-12-13

**Authors:** Diana Quelhas, Chytanya Kompala, Brittney Wittenbrink, Zhen Han, Megan Parker, Myra Shapiro, Shauna Downs, Klaus Kraemer, Jessica Fanzo, Saul Morris, Katharine Kreis

**Affiliations:** 1 Nutrition Innovation, PATH, 2201 Westlake Avenue, Suite 200, Seattle, WA 98121 USA; 2 Johns Hopkins Bloomberg School of Public Health, 615 N Wolfe St, Baltimore, MD 21205 USA; 3 Johns Hopkins Berman Institute of Bioethics, 1809 Ashland Ave, Baltimore, MD 21205 USA; 4 Sight and Life Foundation, PO Box 2116, 4002 Basel, Switzerland; 5 GAIN, Churchill House, 142-146 Old Street, London, EC1V 9BW UK

**Keywords:** Tobacco, Pregnancy, Child growth, Length/height, Small for gestational age, Head circumference

## Abstract

**Background:**

Despite considerable global efforts to reduce growth faltering in early childhood, rates of stunting remain high in many regions of the world. Current interventions primarily target nutrition-specific risk factors, but these have proven insufficient. The objective of this study was to synthesize the evidence on the relationship between active tobacco use during pregnancy and growth outcomes in children under five years of age.

**Methods:**

In this systematic review and meta-analysis, six online databases were searched to identify studies published from January 1, 1980, through October 31, 2016, examining the association between active tobacco use during pregnancy and small-for-gestational age (SGA), length/height, and/or head circumference. Ecological studies were not included. A meta-analysis was conducted, and subgroup analyses were carried out to explore the effect of tobacco dosage.

**Results:**

Among 13,189 studies identified, 210 were eligible for inclusion in the systematic review, and 124 in the meta-analysis. Active tobacco use during pregnancy was associated with significantly higher rates of SGA (pooled adjusted odds ratio [AORs] = 1.95; 95% confidence interval [CI]: 1.76, 2.16), shorter length (pooled weighted mean difference [WMD] = 0.43; 95% CI: 0.41, 0.44), and smaller head circumference (pooled WMD = 0.27; 95% CI: 0.25, 0.29) at birth. In addition, a dose-response effect was evident for all growth outcomes.

**Conclusion:**

Tobacco use during pregnancy may represent a major preventable cause of impaired child growth and development.

**Electronic supplementary material:**

The online version of this article (10.1186/s12889-018-6137-7) contains supplementary material, which is available to authorized users.

## Background

Globally, chronic undernutrition and the resultant growth faltering affect approximately 23% of children under five years of age [1]. The prevalence of stunting, the most common indicator for growth faltering and defined as height-for-age z-score (HAZ) two or more standard deviations below the median of the reference population, is highest in Asia and Africa, where it is estimated to affect 87 million and 59 million children under five years of age, respectively [1]. Chronic undernutrition and growth faltering are associated with long-term health outcomes that span all life stages. These include increased childhood morbidity and mortality, loss of physical growth potential, reduced neurodevelopmental and cognitive function, elevated risk of chronic disease in adulthood, reduced educational attainment, and impaired economic productivity [2, 3].

In 2013, *The Lancet* series on Maternal and Child Nutrition highlighted that beyond a package of ten proven nutrition-specific interventions, the factors contributing to stunting have still not been comprehensively characterized [4]. There is a growing awareness of the complexity of growth faltering and the need to complement nutrition-specific efforts that address the immediate determinants of malnutrition with nutrition-sensitive approaches that address the underlying determinants of malnutrition in order to accelerate progress.

The first 1000 days of life, from conception through age two years, present a critical developmental period, in which most linear growth faltering occurs [3, 5]. Within this timeframe, the gestational period is considered the most critical because linear growth occurs most rapidly in utero. Therefore, pregnancy is an essential window of opportunity in which nutrition-sensitive interventions targeting the mother can have an optimal effect on stunting reduction in the child later in life [3]. In addition to factors associated with the mother’s characteristics and underlying health conditions, there are several external modifiable exposures that may have an important impact on the developing fetus during pregnancy.

Tobacco use by women of reproductive age is highly prevalent in many regions [6], yet it is often understudied and underestimated, especially among vulnerable and remote populations in low-income country settings. In 2015, there were an estimated 933 million daily smokers worldwide, representing 25% of all men and 5.4% of all women [7]. The total number of tobacco users is on the rise, particularly in the developing world, due to population growth, an emerging market for consumption, and a lack of regulations [7]. Without the implementation of reduction strategies, estimated smoking rates will increase to 20% by 2025 among women in low-income countries [6, 8]. Globally, 1.7% of pregnant women smoke and 8.1% of pregnant European women smoke [9]. Almost three-quarters (72.5%) of pregnant women who smoke are daily smokers, and 27.5% of them are occasional smokers. The proportion of women who smoke daily and continue to smoke daily during pregnancy is 52.9%, ranging from 30.6% in the European Region to 79.6% in the Western Pacific Region [9].

In addition to cigarette and pipe smoking, the global tobacco burden encompasses smokeless products such as snuff, chewing products, and other traditional products. More than 300 million people in at least 70 countries use smokeless tobacco products [10]. An analysis of 54 low- and middle-income countries found that in 21 countries, smokeless tobacco was the primary form of tobacco use among pregnant women [11]. Interestingly, there is some geographic overlap of high prevalence of tobacco use by women and high prevalence of stunting in children. This is the case for countries in South Asia such as India and Bangladesh and for certain countries in sub-Saharan Africa.

There is considerable existing evidence linking tobacco use during pregnancy with child measurements such as low birth weight (LBW) [12–19] or small fetal size during gestation [20]. However, these measurements have often lacked an adjustment for gestational age, and therefore the relationship between tobacco use during pregnancy and linear growth faltering has not yet been fully established.

Recent years have seen the emergence of descriptive and inferential studies using gestational growth outcomes that are better indicators of early linear growth faltering than is LBW. Specifically, small-for-gestational age (SGA), most often defined as a fetal/infant length/weight for gestational age below the tenth percentile of the reference population, has been shown to be a useful marker for early linear growth faltering. Three recent studies have established the relationship between SGA and stunted linear growth by five years of age, suggesting that SGA is an important potential precursor of stunting [21–23].

In addition, length at birth, particularly among term births, has been shown to be a strong predictor of height later in life [24]. Also of interest is head circumference, which is reflective of brain size and linked to cognitive function in young children, independent of birth weight [25].

To date, no systematic review has been conducted to characterize the association between tobacco use during pregnancy and linear growth faltering using these indicators. To explore this potential nutrition-sensitive risk factor, we considered it important and timely to review and synthesize the current literature on the relationship between tobacco use during pregnancy and gestational growth outcomes that likely lead to linear growth faltering.

The objective of this systematic review and meta-analysis was therefore to examine the impact of tobacco use during pregnancy on child growth, as measured by SGA, length/ height, and head circumference in children under five years of age.

## Methods

### Search strategy and selection criteria

In this systematic review and meta-analysis, we conducted comprehensive and structured literature searches in CABI Global Health, CINAHL, Embase, Global Index Medicus, PubMed, and Web of Science databases to identify studies published from January 1, 1980, through October 31, 2016, on the association between active tobacco use during pregnancy and child growth. Our search strategies consisted of a combination of Medical Subject Headings (MeSH), free-text words, and words in titles and abstracts, for the exposure population, the exposure of interest, the outcome population, and the outcomes of interest. We limited the search to published studies in English, French, Portuguese, and Spanish. No regional restrictions were imposed, and all study designs except ecological were considered. The detailed search strategy used for PubMed is provided in Additional file 1: Appendix 1. The complete list of all studies included in this systematic review is listed in Additional file 2: Appendix 2.

During the screening process, studies that collected data on active tobacco use as either a dichotomous measure, dosage, and/or frequency at any time during pregnancy based on self-reporting and/or biomarker assessment were included in the review. At least one of the following child growth outcomes was considered, when measured during gestation, at birth, or until five years of age: SGA, length/height, and head circumference (Additional file 3: Table S1). Two definitions for SGA were considered: fetal/infant length/weight for gestational age either below the tenth percentile, or two standard deviations or more below the median, according to a population reference or standard growth curves. We excluded LBW as an outcome during the screening process after verifying that a systematic review focusing on this specific association was being conducted simultaneously. Titles and abstracts were independently screened by two reviewers to identify eligible studies according to the inclusion and exclusion criteria (Additional file 4: Table S2), and conflicts were resolved by a third reviewer. Finally, full texts were independently assessed by the two reviewers.

Data from each study were extracted and synthesized in Microsoft Excel. A quality assessment of all 210 studies included in the systematic review was conducted using a set of 12 criteria adapted from National Institutes of Health quality assessment tools (Additional file 5: Table S3). Case-control studies were scored on a scale of 0 to 26 points based on these criteria, where the quality of studies with 0 to 12 points was considered ‘poor,’,13 to 17 points ‘fair,’ and 18–26 points ‘good.’ All other study types were scored on a scale of 0 to 24, where the quality of studies with 0 to 10 points was considered ‘poor,’ 11 to 15 points ‘fair,’ and 16 to 24 points ‘good.’ In addition, the proportion of studies that met each quality criterion was calculated.

### Data analysis

Meta-analyses using random effects models were conducted in a subset of statistically comparable studies. Only studies that provided sufficient information to compute pooled effect size for the relationship between active tobacco use and child growth outcomes were included in the meta-analysis. Effect measures that did not include measures of significance and/or sample sizes were not included. In the meta-analyses, only studies defining SGA as a birthweight below the 10th percentile, were included. Studies defining SGA as a birthweight below > 2 standard deviations (SD) were therefore excluded. Additionally, for associations between tobacco use and SGA, we only included studies that adjusted their analyses for at least one potential confounder variable, either through multivariate or stratified analyses. For length and head circumference, only studies reporting these outcomes as a continuous measurement in centimeters, were included. Studies reporting outcomes as Z-scores were therefore excluded. For these outcomes, only unadjusted results were reported in the studies, and therefore all such studies were included.

To compute meaningful pooled effect sizes and to understand sources of between-study heterogeneity, we conducted subgroup analyses planned post hoc based on how studies stratified their tobacco exposure variables. The most frequent comparisons were between “never” smoking during pregnancy and either “quitting” smoking anytime during pregnancy, “ever” smoking during pregnancy (meaning smoking any unspecified amount of cigarettes), smoking 1 to 10 cigarettes per day, or smoking more than 10 cigarettes per day. In terms of the child’s age, to maintain comparability, we limited these subgroup analyses to outcome measures taken at birth.

We generated forest plots to summarize the pooled effect size. We report SGA results as pooled adjusted odds ratios (AORs) with 95% confidence intervals (CIs), and length/height and head circumference results, as pooled weighted mean differences (WMDs) with a 95% CI. Heterogeneity was assessed with the *I*^2^ statistic. A sensitivity analysis was done by conducting a meta-regression using as explanatory variables the following study characteristics: study design, quality score, and number of co-variates adjusted for. All analyses were done using STATA version 13.1. (Stata Corporation, College Station, Texas, USA) and R version 3.5.1. (The R Foundation for Statistical Computing, Vienna, Austria).

The PROSPERO ID of this systematic review’s protocol is CRD42016045636, and the PRISMA guidelines for reporting results were followed (Additional file 6: Appendix 3) [26].

### Availability of data and materials

The data files were deposited into the public repository Figshare.com and are labeled ‘Quelhas Data Extraction,’ ‘Quelhas SGA,’ and ‘Quelhas Length and HC.’

## Results

**Fig. 1:**
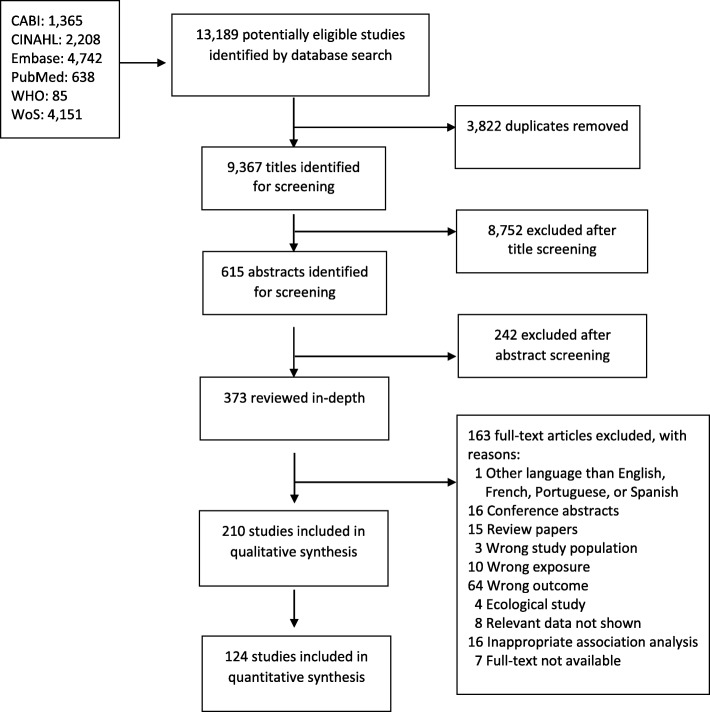
Flow diagram of the study selection process

Among 13,189 citations identified by the search strategy, 406 studies were eligible for full text review. Of these, 210 studies were eligible for inclusion in the systematic review and 124 for inclusion in the meta-analysis (Fig. 1). Our review included 29 case-control studies, 41 cross-sectional studies, 75 prospective cohorts, 42 retrospective cohorts, and 23 with unclear study designs. Sample sizes ranged from 41 to 12,461,312 mother-child pairs, and a total of 26,541,086 pairs were included in all studies. SGA was measured in 125 studies, length/height in 85 studies, head circumference in 77 studies and stunting in 2 studies. These studies were conducted in 43 countries, which were mostly middle- and high-income nations; one multi-country study included countries in Africa and South Asia. Most commonly, tobacco use was defined as cigarette smoking. All studies collected data on active tobacco use from self-reporting by recall during antenatal visits, or retrospectively post-delivery. In addition, 29 of these studies assessed cotinine levels, a specific biomarker of nicotine absorption, present in maternal serum, saliva, urine or hair, and/or infant umbilical cord or meconium specimens. Additional file 3: Table S1 summarizes the characteristics of each study.

All meta-analyses focus on self-reported cigarette smoking as the only mode of tobacco use and on growth outcomes measured at birth.

### Small for gestational age at birth

**Fig. 2:**
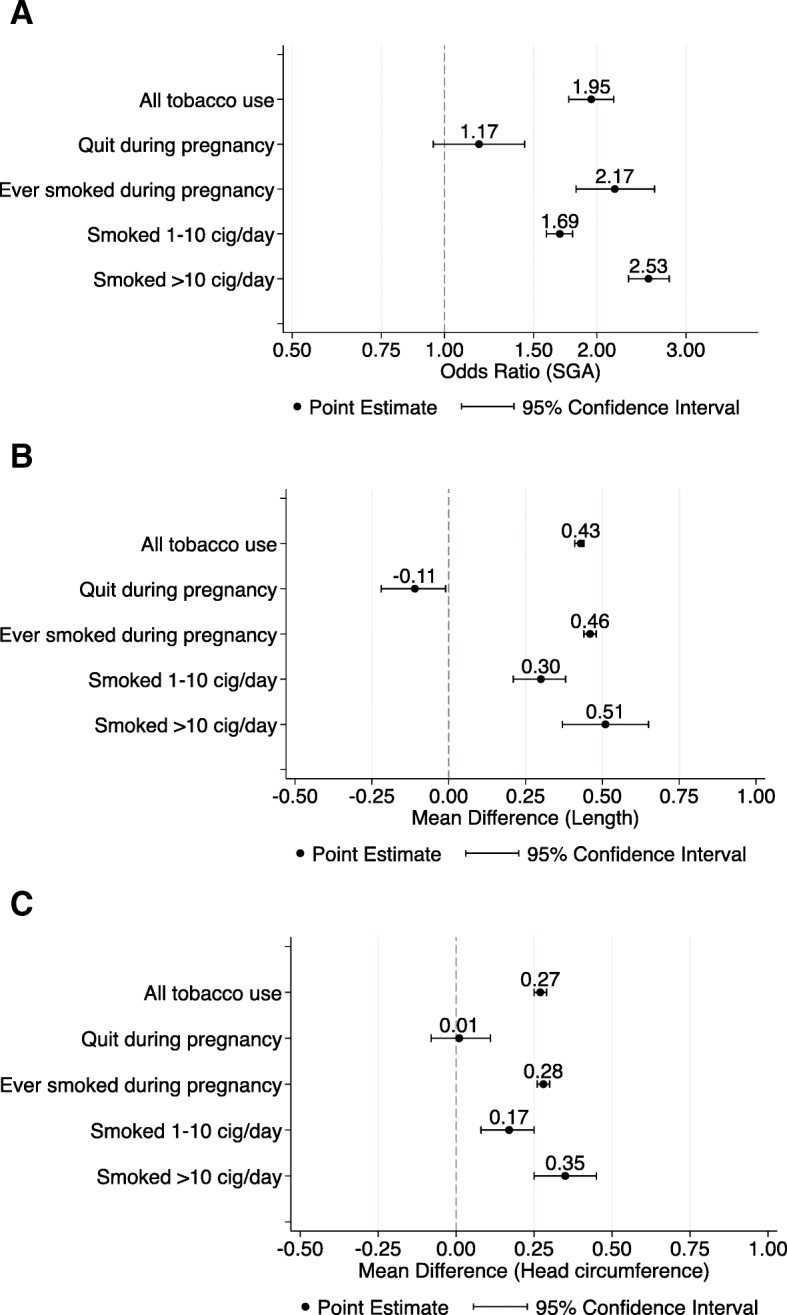
Summary plots for the overall and subgroups of tobacco use on each growth outcome at birth: **a**. SGA; **b**. Length; **c**. Head circumference

**Fig. 3:**
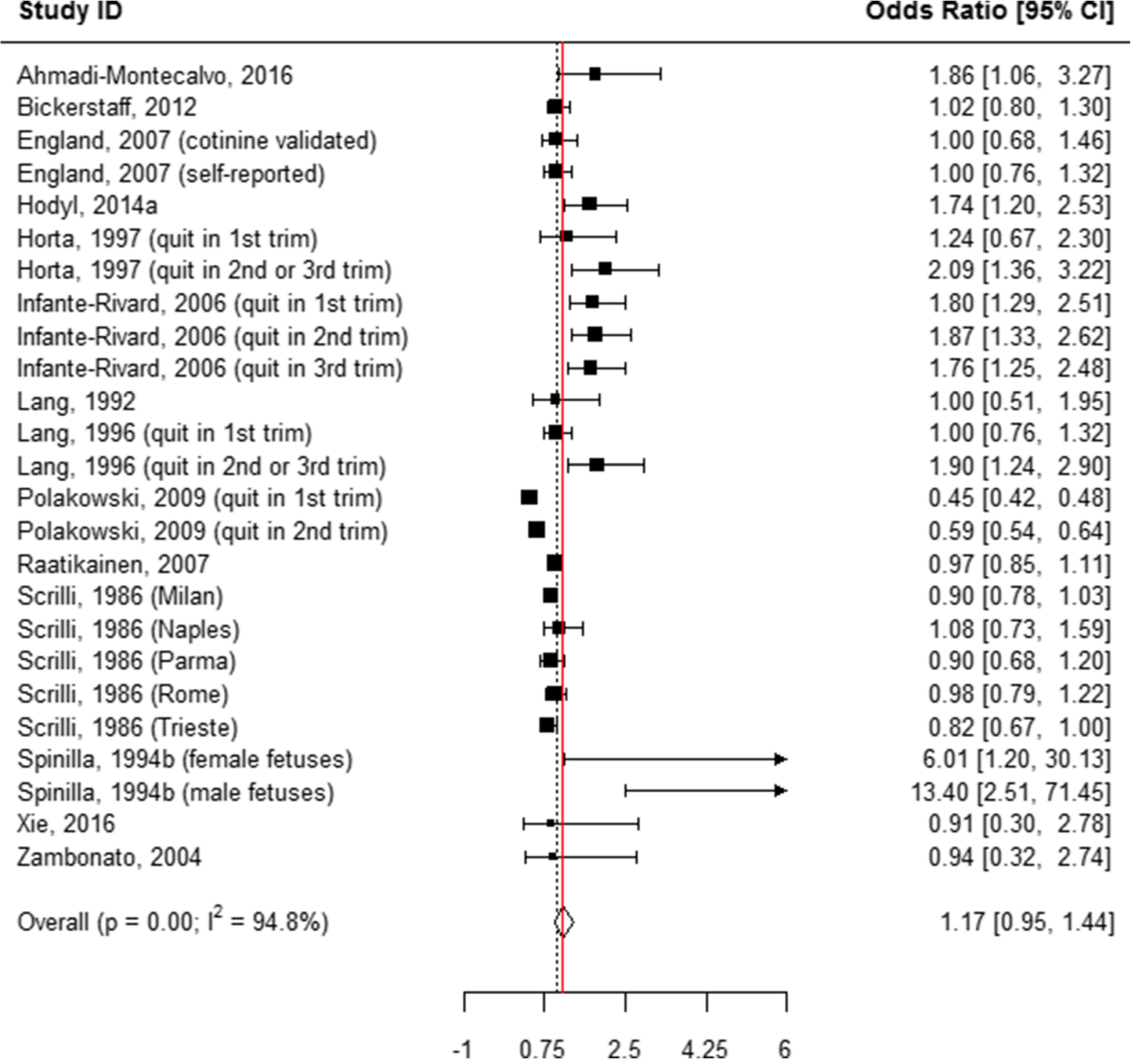
Effect of active tobacco use during pregnancy on SGA: Quitters. Reference group is Non-smokers

**Fig. 4:**
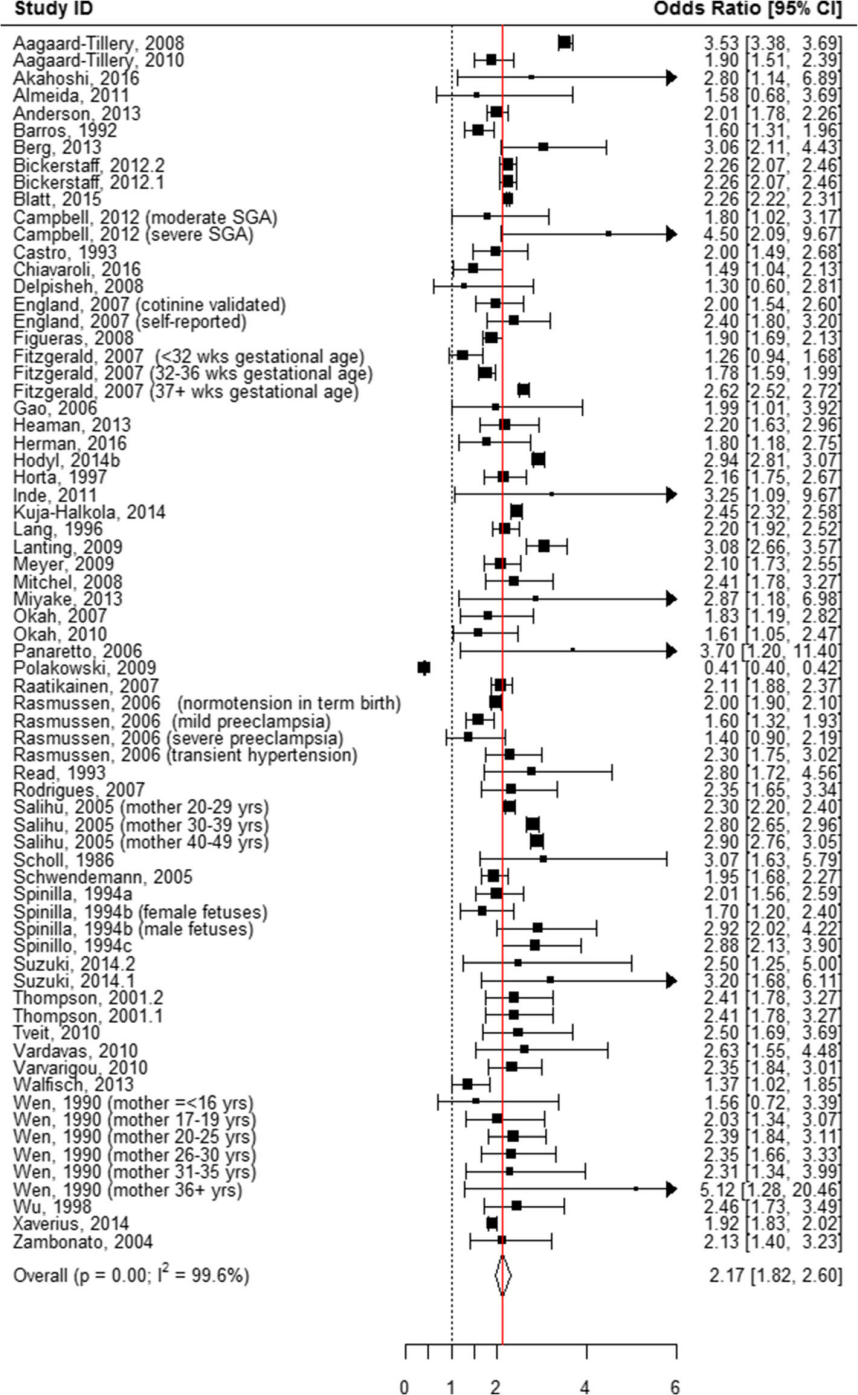
Effect of active tobacco use during pregnancy on SGA: Ever smoked during pregnancy. Reference group is Non-smokers

**Fig. 5:**
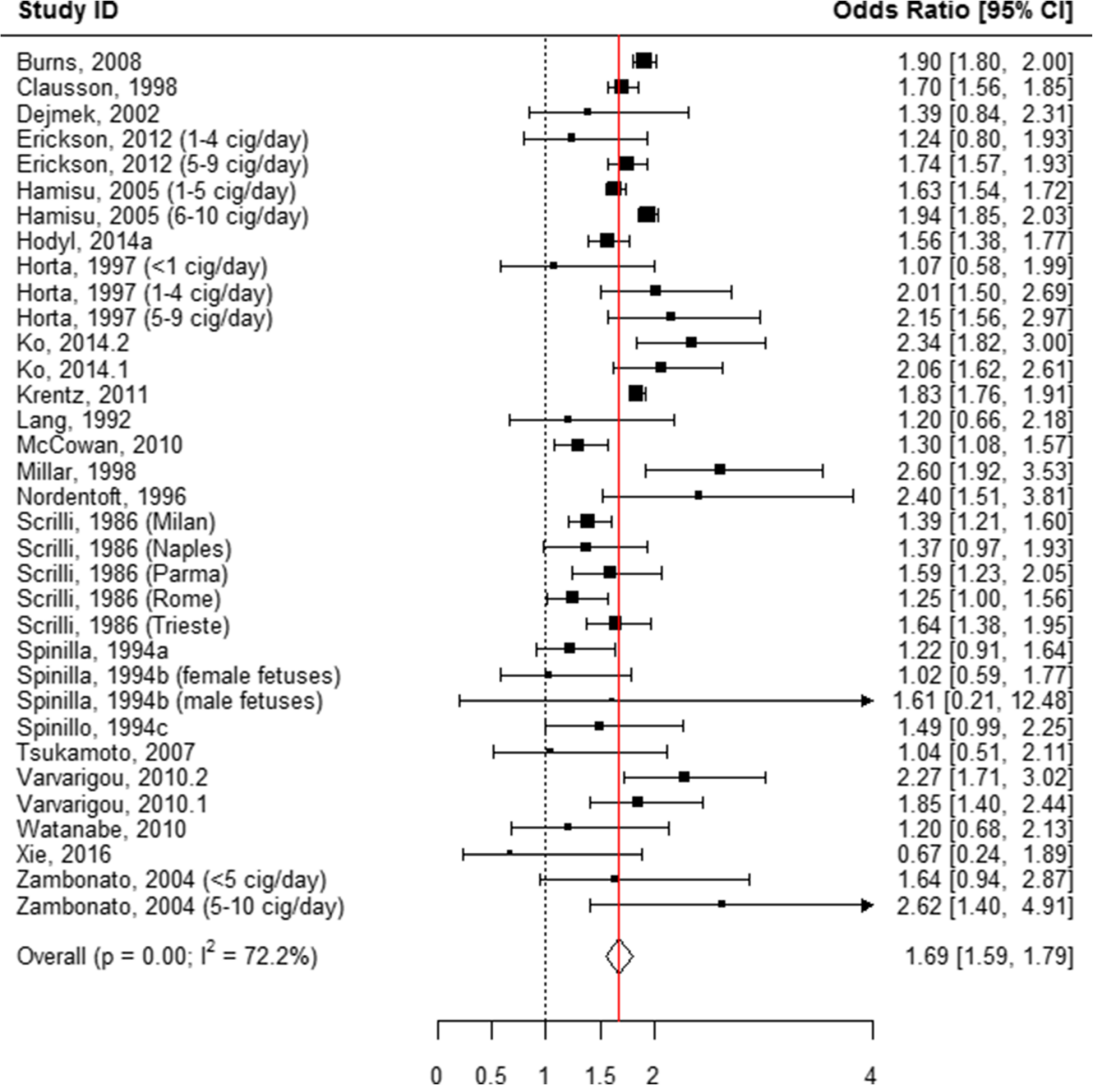
Effect of active tobacco use during pregnancy on SGA: 1–10 cigarettes/day. Reference group is Non-smokers

**Fig. 6:**
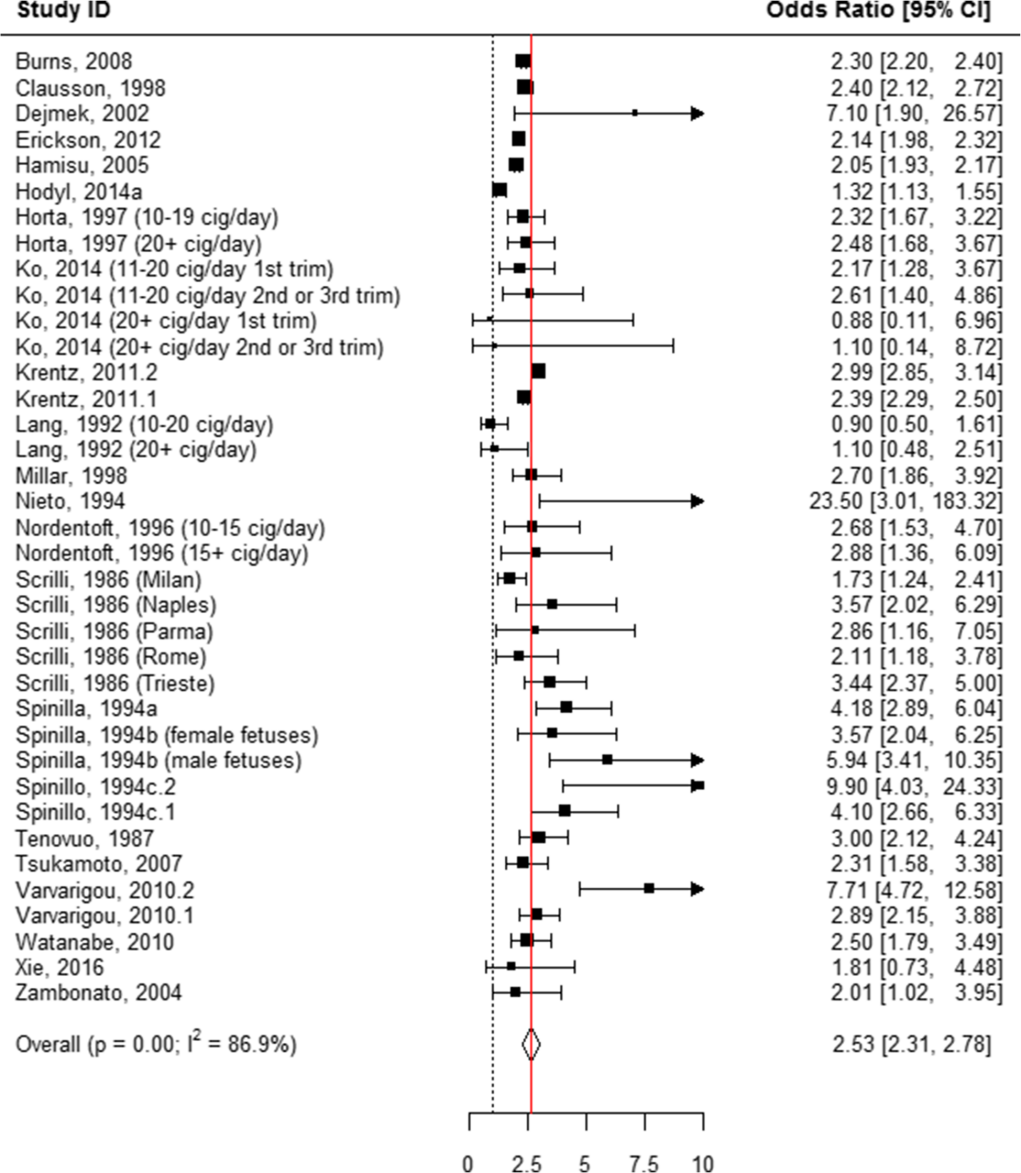
Effect of active tobacco use during pregnancy on SGA: > 10 cigarettes/day. Reference group is Non-smokers

The following mutually exclusive categories of reported smoking were considered: smoked 1 to 10 cigarettes per day, smoked > 10 cigarettes per day, “ever” smoked during pregnancy, and “quit smoking” anytime during pregnancy. When all of these categories were pooled, smoking during pregnancy was associated with an odds of 1.95 of having an SGA infant as compared to women who never smoked during pregnancy (AOR = 1.95, 95% CI 1.76, 2.16; Table 1, Additional file 7: Figure S1, Fig. 2). When the association between the quantity of tobacco used and the risk of SGA was evaluated in the subgroup analyses, quitting smoking at any time during pregnancy showed no association with having an SGA infant (AOR = 1.17, 95% CI 0.95, 1.44; Table 1, Figs. 2 and 3). “Ever” smoking during pregnancy was strongly associated with SGA (AOR = 2.17, 95% CI 1.82, 2.60; Table 1, Figs. 2 and 4) as compared to “never” smoking during pregnancy. When the amount of tobacco consumption was specified, women who smoked up to 10 cigarettes daily were 1.69 times more likely to give birth to SGA infants as compared to women who did not smoke any cigarettes during pregnancy (AOR = 1.69, 95% CI 1.59, 1.79; Table 1, Figs. 2 and 5). Women who smoked more than 10 cigarettes daily were 2.53 times more likely to give birth to SGA infants as compared to those who did not smoke any cigarettes during pregnancy (AOR = 2.53, 95% CI 2.31, 2.78; Table 1, Figs. 2 and 6).

**Table 1:** Meta-analysis summary pooled estimates for the effect of overall and subgroups of tobacco use on each growth outcome at birth

	No. of studies	No. of associations	Pooled estimates	Heterogeneity		
				X^2^	*P*	*I* ^2^
SGA at birth			AOR ^a^ (95% CI)			
All tobacco use (all of the below)	71	168	1.95 (1.76, 2.16)	21253.97	<0.001	99.2%
Quit during pregnancy	14	25	1.17 (0.95, 1.44)	458.62	<0.001	94.8%
Ever smoked during pregnancy	52	70	2.17 (1.82, 2.60)	17475.92	<0.001	99.6%
Smoked 1-10 cigarettes/day	22	34	1.69 (1.59, 1.79)	118.70	<0.001	72.2%
Smoked >10 cigarettes/day	23	37	2.53 (2.31, 2.78)	267.16	<0.001	86.9%
Length at birth			WMD ^b^ (95% CI)			
All tobacco use (all of the below)	47	72	0.43 (0.41, 0.44)	1157.71	<0.001	93.9%
Quit during pregnancy	4	4	-0.11 (-0.22, -0.01)	2.02	0.569	0.0%
Ever smoked during pregnancy	40	44	0.46 (0.44, 0.48)	969.73	<0.001	95.6%
Smoked 1-10 cigarettes/day	7	7	0.30 (0.21, 0.38)	15.56	0.016	61.4%
Smoked >10 cigarettes/day	8	10	0.51 (0.37, 0.65)	34.87	<0.001	78.6%
Head circumference at birth			WMD ^b^ (95% CI)			
All tobacco use (all of the below)	43	71	0.27 (0.25, 0.29)	707.87	<0.001	90.1%
Quit during pregnancy	5	5	0.01 (-0.08, 0.11)	3.25	0.517	0.0%
Ever smoked during pregnancy	37	41	0.28 (0.26, 0.30)	631.13	<0.001	93.7%
Smoked 1-10 cigarettes/day	7	7	0.17 (0.08, 0.25)	5.04	0.539	0.0%
Smoked >10 cigarettes/day	8	11	0.35 (0.25, 0.45)	24.40	0.010	54.9%

^a^AOR, Adjusted odds ratio

^b^WMD, Weighted mean difference

A high level of between-study heterogeneity was observed when pooling all studies measuring the association between tobacco use during pregnancy and SGA (I^2^ = 99.2%, *p* < 0.001) (Table 1). Study quality (*p* = 0.06), study design (*p* = 0.98), and the number of co-variates adjusted for (*p* = 0.83) had no influence on heterogeneity, as indicated by a meta-regression. However, when these factors were examined through subgroup analyses across all tobacco use categories, studies with a “fair” quality score and case-control studies were more likely to show stronger associations between tobacco use during pregnancy and SGA. Importantly, the strong association between women who smoked more than 10 cigarettes daily and SGA remained unchanged irrespective of the number of covariates that were adjusted for in the model.

### Length at birth

**Fig. 7:**
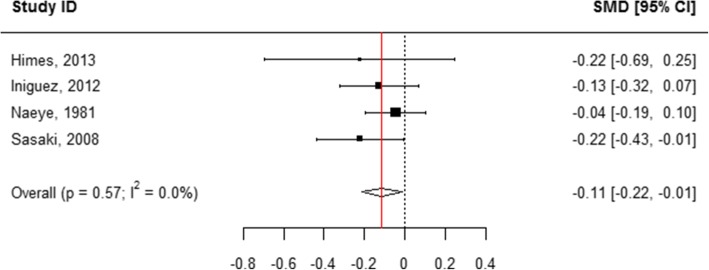
Effect of active tobacco use during pregnancy on length (cm): Quitters. Reference group is Non-smokers

**Fig. 8:**
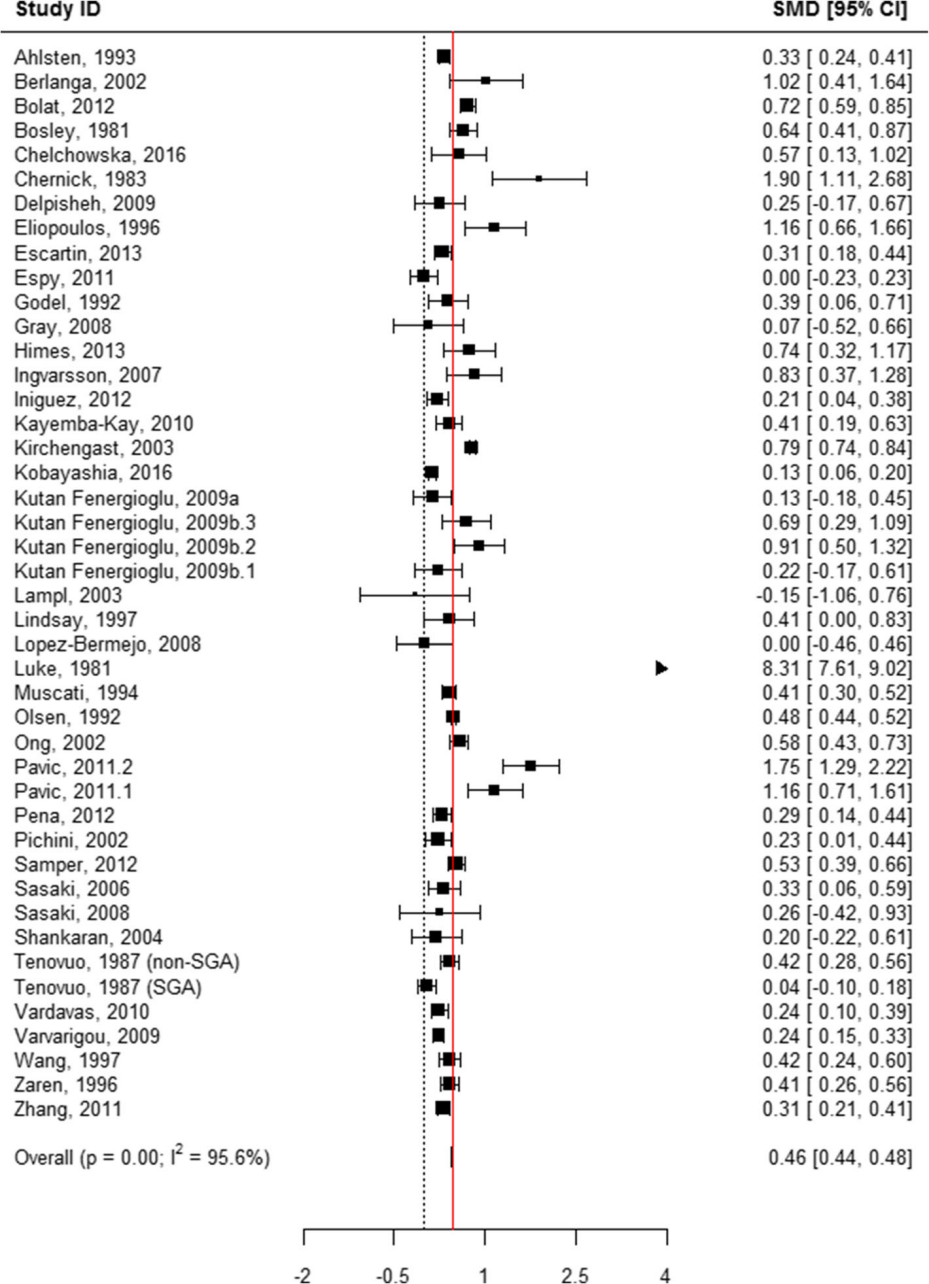
Effect of active tobacco use during pregnancy on length (cm): Ever smoked during pregnancy. Reference group is Non-smokers

**Fig. 9:**
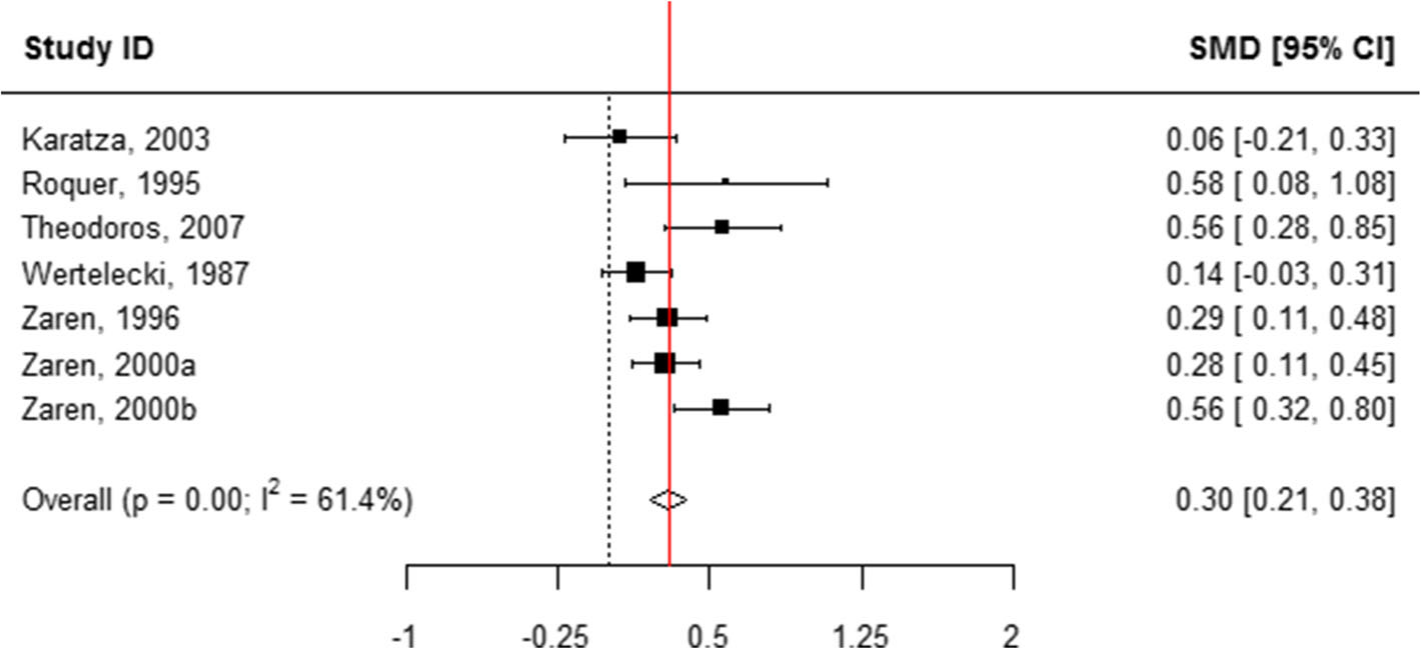
Effect of active tobacco use during pregnancy on length (cm): 1–10 cigarettes/day. Reference group is Non-smokers

**Fig. 10:**
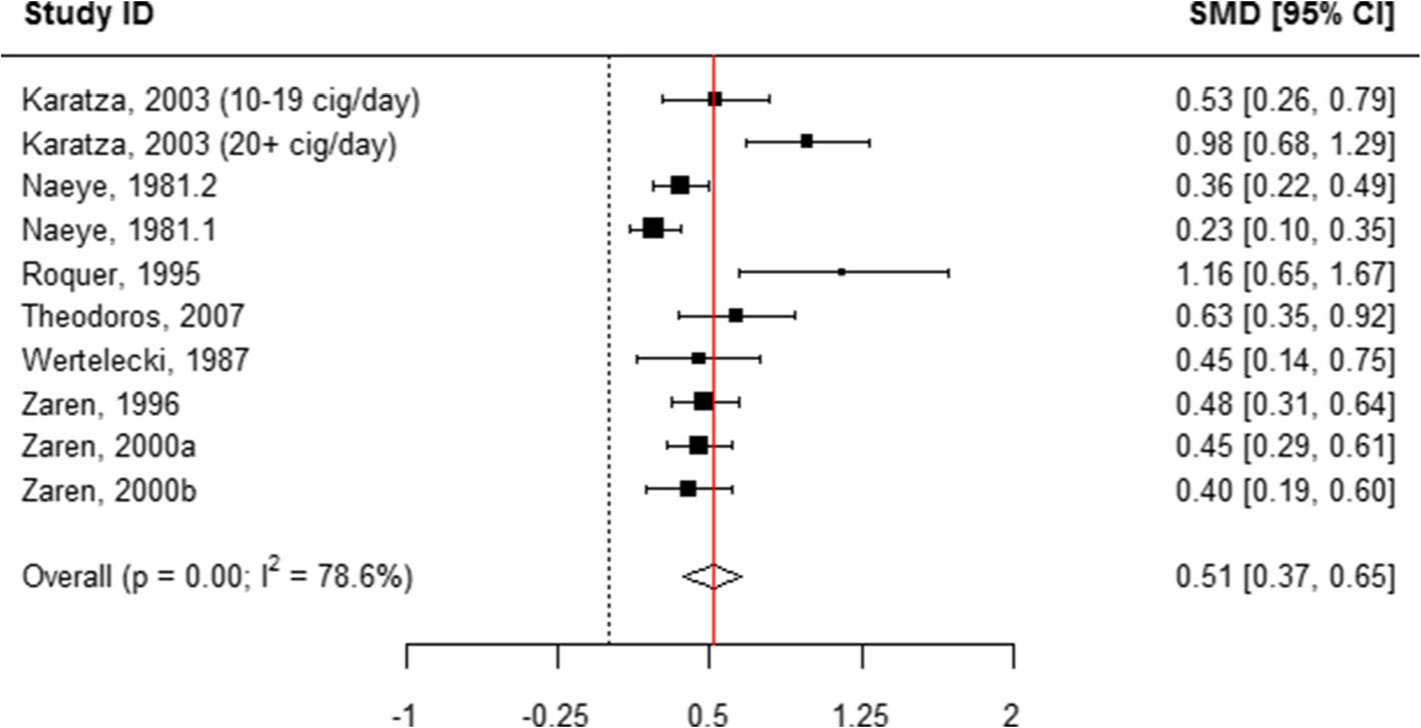
Effect of active tobacco use during pregnancy on length (cm): > 10 cigarettes/day. Reference group is Non-smokers

When all categories of smoking were pooled, children born to mothers who actively used tobacco during pregnancy were 0.43 cm shorter in length at birth compared with those born to mothers who did not smoke (WMD = 0.43, 95% CI 0.41, 0.44; Table 1, Additional file 8: Figure S2 and Fig. 2). A subgroup analysis of the association between the quantity of tobacco used and the child’s length at birth found that mothers who quit smoking at any time during pregnancy had infants who were 0.11 cm longer than mothers who did not report smoking (WMD = − 0.11, 95% CI -0.22, − 0.01; Table 1, Figs. 2 and 7). “Ever” smoking during pregnancy was associated with infants who were 0.46 cm shorter at birth (WMD = 0.46, 95% CI 0.44, 0.48; Table 1, Figs. 2 and 8). When the tobacco amount was specified, women who smoked up to 10 cigarettes daily had infants who were 0.30 cm shorter as compared to those who did not smoke any cigarettes during pregnancy (WMD = 0.30, 95% CI 0.21,0.38; Table 1, Figs. 2 and 9). Women who smoked more than 10 cigarettes daily had infants who were 0.51 cm shorter as compared to those who did not smoke during pregnancy (WMD = 0.51, 95% CI 0.37, 0.65; Table 1, Figs. 2 and 10).

### Head circumference at birth

**Fig. 11:**
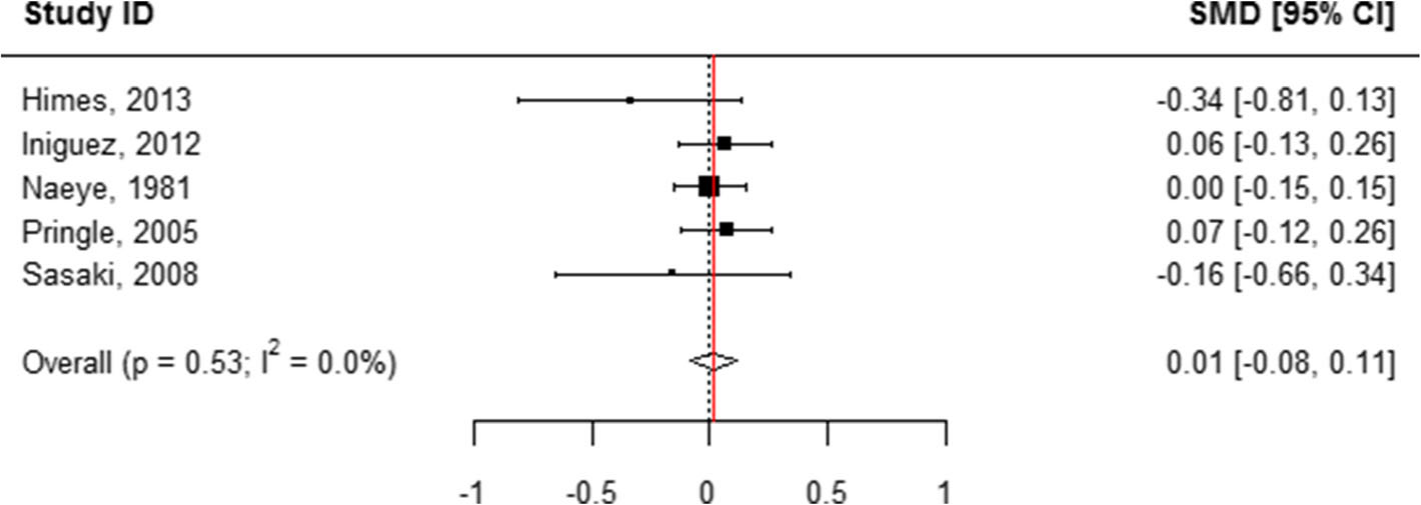
Effect of active tobacco use during pregnancy on head circumference (cm): Quitters. Reference group is Non-smokers

**Fig. 12:**
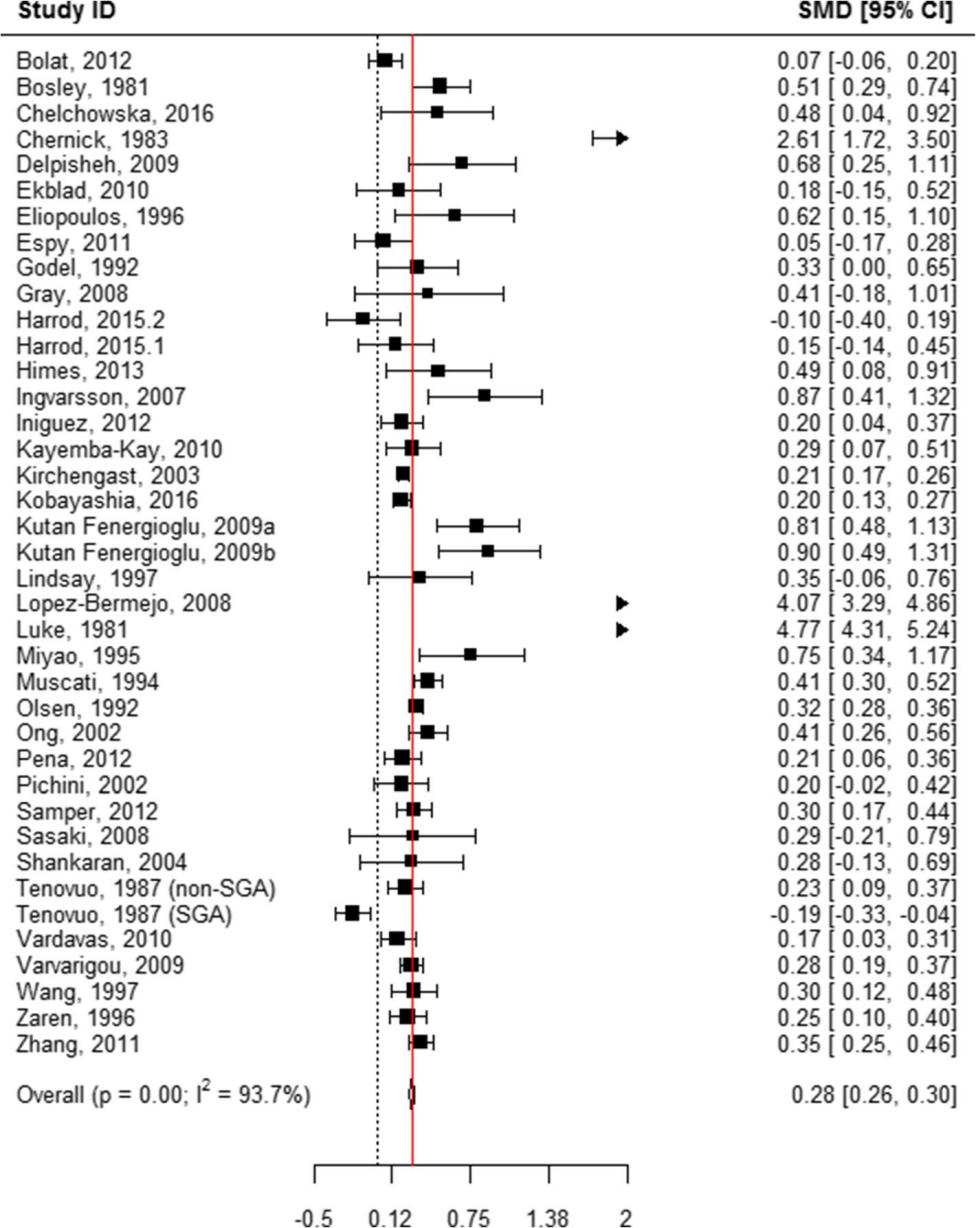
Effect of active tobacco use during pregnancy on head circumference (cm): Ever smoked during pregnancy. Reference group is Non-smokers

**Fig. 13:**
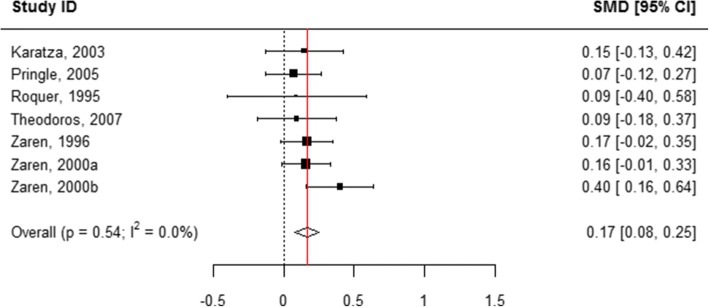
Effect of active tobacco use during pregnancy on head circumference (cm): 1–10 cigarettes/day. Reference group is Non-smokers

**Fig. 14:**
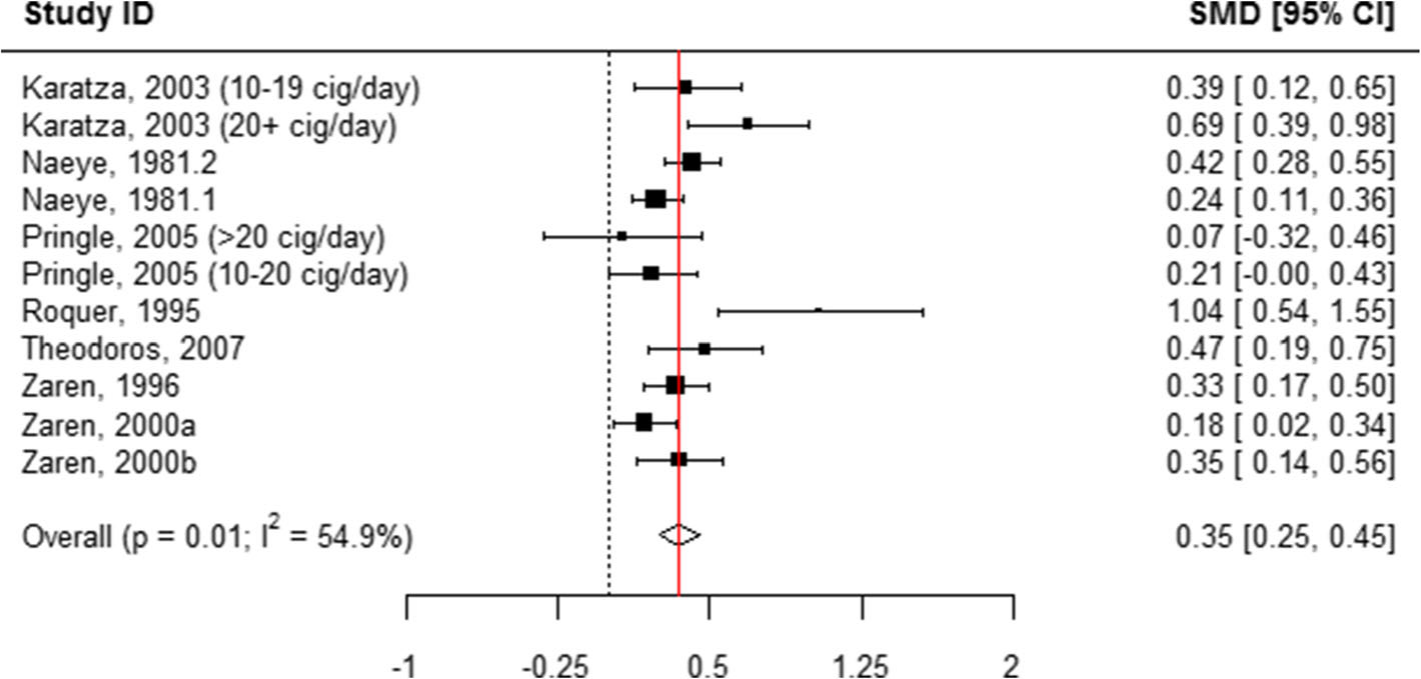
Effect of active tobacco use during pregnancy on head circumference (cm): > 10 cigarettes/day. Reference group is Non-smokers

When all categories of smoking were pooled, children born to mothers who actively used tobacco during pregnancy had an average head circumference that was 0.27 cm smaller than that of infants born to mothers who did not smoke (WMD = 0.27, 95% CI 0.25, 0.29; Table 1, Additional file 9: Figure S3, Fig. 2). Among mothers who reported quitting smoking during pregnancy, no statistically significant difference was observed in child head circumference in comparison to mothers who reported not smoking (WMD = 0.01, 95% CI -0.08, 0.11; Table 1, Figs. 2 and 11). “Ever” smoking during pregnancy was associated with 0.28 cm smaller head circumference at birth (WMD = 0.28, 95% CI 0.26, 0.30; Table 1, Figs. 2 and 12). When the amount of tobacco consumption was specified, women who smoked up to 10 cigarettes daily give birth to infants whose average head circumference was 0.17 cm smaller than that of infants of women who did not smoke during pregnancy (WMD = 0.17, 95% CI 0.08, 0.25; Table 1, Figs. 2 and 13). Women who smoked more than 10 cigarettes daily gave birth to infants whose average head circumference was 0.35 cm smaller than that of infants whose mothers did not smoke during pregnancy (WMD = 0.35, 95% CI 0.25, 0.45; Table 1, Figs. 2 and 14).

### Quality assessment

**Fig. 15:**
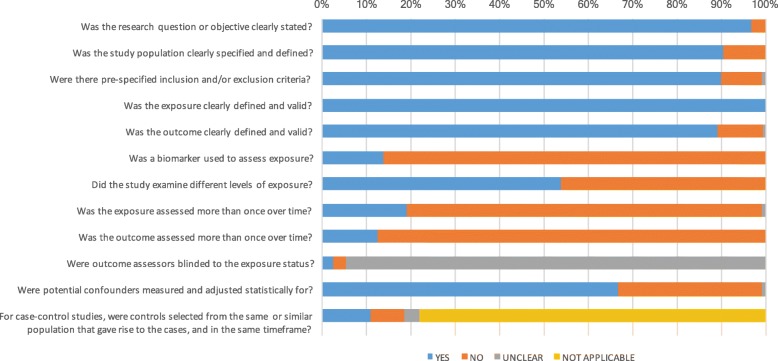
Quality assessment of studies included in the systematic review: proportion of studies that meet each of the quality criteria

Based on our quality assessment of individual studies, 80 studies were judged as “good,” 118 as “fair,” and 12 as “poor” quality (Additional file 3: Table S1). Figure 15 shows the proportion of studies meeting each quality criterion. Nearly all studies had clearly defined research questions, exposures, and outcomes. Only 67% of studies controlled for one or more confounders in their analysis.

## Discussion

To our knowledge, this is the first study to review and synthesize evidence on the association between tobacco use during pregnancy and SGA, length, and head circumference in children under five years of age.

Our search strategy for this systematic review was designed to include studies from all geographical regions, by using international databases and including studies published in four languages. We aimed to include all forms of tobacco use, by using more than 40 terms for conventional as well as traditional smoked and smokeless tobacco used in different countries. We also included terms to include measurement of growth outcomes in children from the gestation period up to five years of age.

As such, our expectation was that we would identify studies from regions with overlapping high prevalence of child stunting and tobacco use among women of reproductive age. However, despite the large number of studies included, our compilation did not represent those regions of interest or the diversity of tobacco products available. Again, despite the inclusive age search terms, very few studies included anthropometric measurements taken during gestation or beyond the moment of birth.

Based on the studies included in our meta-analysis, focused only on measurements taken at birth, tobacco use during pregnancy has a negative impact on all growth outcomes considered. These findings suggest that prenatal exposure to tobacco could potentially lead to stunting and compromise brain development during early childhood and perhaps later in life.

Additionally, we observed a clear dose-response relationship across all three growth outcomes such that increased consumption of tobacco further exacerbated growth restriction. Previous studies examining the effect of maternal smoking on LBW and perinatal and neonatal mortality also found a clear dose-response gradient [27, 28]. This finding may have practical relevance for efforts to reduce stunting, because considerably reducing the number of cigarettes smoked or quitting early in pregnancy may significantly improve gestational growth outcomes measured at birth.

It has been suggested that restrictions to linear growth during gestation may be compensated for with effective interventions implemented during the early developmental years [29, 30]. Interestingly, the six studies that tracked child length and head circumference for longer periods suggested a slight catch-up growth trend. This was illustrated by a steadily decreasing difference between the tobacco-exposed children and non-exposed children between 2 and 5 years of age [31–36].

The observed associations between smoking during pregnancy and impaired linear growth may reflect several known biological mechanisms that may lead directly or indirectly to suboptimal development of the fetus and young child. Tobacco use results in oxidative stress in the placenta, leading to restricted flow of oxygen and nutrients to fetus [37], and in reduced folate levels, which affects child development and growth [38]. Additionally, epigenetic modification in utero occurs and manifests in DNA methylation and disruption of human growth plate chondrocytes, delaying skeletal growth [39–41].

Our systematic review highlights the limitations of the available data and a need for future inquiry. The fact that only 29 studies used biomarkers to examine tobacco use may have resulted in an important misclassification of smoking status and thus an underestimation of true tobacco use [42]. Future research should rely more on biomarkers than on self-reported data. Further, most studies reported only on cigarette smoking despite the inclusion of 40 search terms for different types of smoked and smokeless tobacco products. The impact of smokeless tobacco is likely to be under-studied and under-reported, leaving out information on this association from our analysis.

It is also essential that future studies control for known and potential confounding factors to more confidently distinguish the attributable effect of maternal tobacco use on child growth from other factors influencing growth during the first 1000 days of life, including maternal physical and mental health, education level, and socio-economic status [43]. The effect of tobacco exposure before conception, after birth, during lactation, or co-exposure to environmental tobacco smoke should be explored in further research [44]. Longer follow-up periods will also allow researchers to assess trends over time to better understand the long-term impact. Further, for the purpose of assessing long-term growth, LBW is insufficient, and measures such as SGA, HAZ, and potentially HAD (height-for-age difference), which account for age, should be prioritized. Importantly, more research is needed in low- and middle-income countries, where the burden of stunting is highest and tobacco use in on the rise.

Tobacco use has historically been understood as a general public health concern that causes diseases such as cancer, cardiovascular disease, chronic obstructive pulmonary disease, and hypertension. Yet our findings suggest the need for a shift in perception among the public health community. Tobacco use is not only a risk behavior for the general population but also a potential cause of impaired child growth and development. Given the heavy global burden of stunting that hinders individuals, families, communities, and even nations from reaching their full potential, policy and programmatic efforts to optimize child growth and development should consider tobacco use as an important determinant.

Tackling tobacco prevention and cessation, especially among women, is challenging. Tobacco rates are rising in the developing world for several reasons. Tobacco, in all its forms, is an addictive substance, and the lack of public health awareness in low-resource settings means women may not know the risks associated with its use. Behavior change communication, coupled with tobacco cessation support, is essential for developing targeted and culturally appropriate approaches to reducing tobacco use among reproductive-aged women. Market-based approaches targeting the tobacco industry, such as taxes and advertising regulations, should also be considered. Indeed, stunting may be attributed to the presence of additive or multiplicative risk factors. With this in mind, it is important to put in place better systems for tracking tobacco use among pregnant women, and to design complementary and collaborative public health interventions that target children identified as already being at risk for growth faltering due to in-utero exposure to tobacco.

The global health community has already made strides in addressing tobacco use through the development of the 2005 World Health Organization Framework Convention on Tobacco Control, a treaty that obligates 181 signatory countries to implement measures to reduce tobacco use. However, tobacco control strategies have been implemented to various degrees around world, and if additional steps are not taken, tobacco use is projected to rise in low-income nations.

## Conclusion

Tobacco use during pregnancy has a negative impact on all growth outcomes considered in this systematic review and meta-analysis: SGA, length and head circumference at birth. These findings suggest that prenatal exposure to tobacco could potentially lead to stunting and compromise brain development during early childhood and perhaps later in life.

Policy and programmatic efforts to optimize child growth and development should consider tobacco use as an important determinant.

## Additional files

Additional file 1: Appendix 1. Search strategy used for PubMed. Description of data: Appendix 1 includes the search terms for the exposure population, exposures of interest, outcome population, and outcomes of interest. (DOCX 16 kb)

Additional file 2: Appendix 2. List of references of studies included in the systematic review. Description: The complete list of studies included in the systematic review. (DOCX 34 kb)

Additional file 3: **Table S1.** Characteristics of studies included in the systematic review and meta-analysis. Description of data: A complete list of the studies included in the systematic review and meta-analysis. The data included shows each study’s location, design, sample size, growth outcome, and quality of research. (DOCX 35 kb)

Additional file 4: **Table S2.** Inclusion and exclusion criteria for selecting studies for the systematic review and meta-analysis. Description of data: A complete list of inclusion and exclusion criteria used to select the studies for the systematic review and meta-analysis. (DOCX 19 kb)

Additional file 5: **Table S3.** Quality assessment criteria. Description of data: Twelve quality assessment criteria standards and the number of points assigned to each. (DOCX 18 kb)

Additional file 6: Appendix 3. PRISMA 2009 Checklist. Description of data: A checklist indicating where different topics in each section (e.g. eligibility criteria in methods section) are found within the greater document. (DOC 64 kb)

Additional file 7: **Figure S1.** Effect of all tobacco use during pregnancy on SGA at birth. Description of data: A forest plot illustrating the effect of all types of tobacco exposure during pregnancy on SGA at birth as indicated by the Odds Ratio and 95% CI. (PNG 73 kb)

Additional file 8: **Figure S2.** Effect of all tobacco use during pregnancy on length at birth. Description of data: A forest plot illustrating the effect all types of tobacco use and exposure during pregnancy on length at birth as indicated by standardized mean differences (95% CI). (PNG 30 kb)

Additional file 9: **Figure S3.** Effect of all tobacco use during pregnancy on head circumference at birth. Description of data: A forest plot illustrating the effect of all tobacco use during pregnancy on head circumference at birth as indicated by standardized mean differences (95% CI). (PNG 29 kb)

## Abbreviations

AOR: Adjusted odds ratio
CI: Confidence interval
CINAHL: Cumulative Index to Nursing and Allied Health Literature
EMBASE: Excerpta Medica Database
HAD: Height-for-age difference
HAZ: Height-for-age Z-score
LBW: Low birth weight
MEDLINE: Medical Literature Analysis and Retrieval System Online
MeSH: Medical Subject Headings
PRISMA: Preferred Reporting Items for Systematic Reviews and Meta-Analyses
SD: Standard deviation
SGA: Small for gestational age
WMD: Weighted mean difference
