# Supplementary material for: The association between active tobacco use during pregnancy and growth outcomes of children under five years of age: a systematic review and meta-analysis
Source: BMC Public Health. 2018 Dec 13;18:1372. doi: 10.1186/s12889-018-6137-7 (PMC6293508; doi:10.1186/s12889-018-6137-7)
Supplement: Supplementary file 1 — Appendix 1. Search strategy used for PubMed. Description of data: Appendix 1 includes the search terms for the exposure population, exposures of interest, outcome population, and outcomes of interest. (DOCX 16 kb) [file 12889_2018_6137_MOESM1_ESM.docx]

**Additional file 1: Appendix 1.** Search strategy used for PubMed

Search terms for the exposure population:

pregnancy[MeSH] OR pregnan*[TIAB] OR gestation*[TIAB] OR prenatal[TIAB] OR perinatal[TIAB] OR maternal[TIAB] OR gravid[TIAB]

Search terms for the exposure of interest:

tobacco[MeSH] OR tobacco use[MeSH] OR tobacco products[MeSH] OR tobacco, smokeless[MeSH] OR tobacco[TIAB] OR tobacco consumption[TIAB] OR smoke*[TIAB] OR tobacco product*[TIAB] OR cigar*[TIAB] OR smokeless tobacco[TIAB] OR chew* tobacco[TIAB] OR dissolvable tobacco[TIAB] OR pipe[tiab] OR chew*[tiab] OR snus[tiab] OR dissolvable*[tiab] OR hookah[tiab] OR gutkha[TIAB] OR dip* tobacco[TIAB] OR pipe tobacco[TIAB] OR waterpipe[TIAB] OR water pipe[TIAB] OR nicotine[TIAB] OR beedi[TIAB] OR bidi[TIAB] OR bidis[TIAB] OR chimo[TIAB] OR dokha[TIAB] OR gutkha[TIAB] OR gutka[TIAB] OR hookah[TIAB] OR iqmik[TIAB] OR itabi[TIAB] OR khaini[TIAB] OR kilaiku[TIAB] OR kizami[TIAB] OR kretek[TIAB] OR ligwayi[TIAB] OR makla[TIAB] OR mbaki[TIAB] OR midwakh[TIAB] OR mishri[TIAB] OR muassel[TIAB] OR nargile[TIAB] OR narghile[TIAB] OR narjila[TIAB] OR arguile[TIAB] OR nass[TIAB] OR naswar[TIAB] OR paan[TIAB] OR qalyan[TIAB] OR shisha[TIAB] OR sigara[TIAB] OR snefu[TIAB] OR snuff[TIAB] OR snus[TIAB] OR tambook[TIAB] OR tumbako[TIAB] OR tumbaku[TIAB] OR ubugoro[TIAB] OR ugoro[TIAB] OR zarda[TIAB]

Search terms for the outcome population:

child, preschool[MeSH] OR infant[MeSH] OR infant, newborn[MeSH] OR fetus[MeSH] OR child*[TIAB] OR infant*[TIAB] OR newborn infant[TIAB] OR babies[TIAB] OR baby[TIAB] OR toddler*[TIAB] OR preschool child*[TIAB] OR preschooler[TIAB] OR under five[TIAB] OR pediatrics[MeSH] OR pediatric*[TIAB] OR paediatric*[TIAB] OR peadiatric*[TIAB] OR fetus[TIAB] OR foetus[TIAB] OR fetal[TIAB] OR foetal[TIAB]

Search terms for the outcomes of interest:

growth disorders[MeSH] OR fetal growth retardation[MeSH] OR infant, low birth weight[MeSH] OR growth disorder*[TIAB] OR stunting[TIAB] OR stunted growth[TIAB] OR growth outcomes[TIAB] OR fetal growth retardation[TIAB] OR foetal growth retardation[TIAB] OR fetal growth restriction[TIAB] OR foetal growth restriction[TIAB] OR fgr[TIAB] OR intrauterine growth restriction[TIAB] OR intrauterine growth retardation[TIAB] OR iugr[TIAB] OR small for gestational age[TIAB] OR sga[TIAB] OR low birth weight infant[TIAB] OR low birth weight[TIAB] OR lbw[TIAB] OR head circumference*[TIAB] OR anthropometr*[TIAB]
