# Supplementary material for: The association between active tobacco use during pregnancy and growth outcomes of children under five years of age: a systematic review and meta-analysis
Source: BMC Public Health. 2018 Dec 13;18:1372. doi: 10.1186/s12889-018-6137-7 (PMC6293508; doi:10.1186/s12889-018-6137-7)
Supplement: Supplementary file 2 — Appendix 2. List of references of studies included in the systematic review. Description: The complete list of studies included in the systematic review. (DOCX 34 kb) [file 12889_2018_6137_MOESM2_ESM.docx]

**Additional file 2: Appendix 2.** List of references of studies included in the systematic review

1. Ahmadi-Montecalvo H, Haile ZT, Umer A, Chertok IR. Adolescent Pregnancy and Smoking in West Virginia: Pregnancy Risk Assessment Monitoring System (PRAMS) 2005-2010. Matern Child Health J. 2016;(PG-).
2. England LJ, Levine RJ, Mills JL, Klebanoff MA, Yu KF, Cnattingius S. Adverse pregnancy outcomes in snuff users. Am J Obstet Gynecol. 2003;189(4 PG-939-943):939–43.
3. Harrison GG, Branson RS, Vaucher YE. Association of maternal smoking with body composition of the newborn. Am J Clin Nutr. 1983;38(5 PG-757-762):757–62.
4. Iniguez C, Ballester F, Amoros R, Murcia M, Plana A, Rebagliato M. Active and passive smoking during pregnancy and ultrasound measures of fetal growth in a cohort of pregnant women. J Epidemiol Community Heal. 2012;66(6 PG-563-570):563–70.
5. Lang JM, Lieberman E, Cohen A. A comparison of risk factors for preterm labor and term small-for-gestational-age birth. Epidemiology. 1996;7(4 PG-369-376):369–76.
6. Nunes RD, Campos ACC de. Avaliação do hábito tabágico e fatores associados ao tabagismo na gestação Evaluation of smoking habit and its associated factors in pregnancy. ACM arq catarin med. 2016;44(3 PG-23-36):23–36.
7. Ocallaghan MJ, Harvey JM, Tudehope D, Gray PH. Aetiology and classification of small for gestational age infants. J Paediatr Child Health. 1997;33(3 PG-213-218):213–8.
8. Ahlsten G, Cnattingius S, Lindmark G. Cessation of smoking during pregnancy improves foetal growth and reduces infant morbidity in the neonatal period: A population-based prospective study. Acta Paediatr. 1993;82(2 PG-177-181):177–81.
9. Barros FC, Huttly SRA, Victora CG, Kirkwood BR, Vaughan JP. Comparison of the causes and consequences of prematurity and intrauterine growth retardation: a longitudinal study in southern Brazil. Pediatrics. 1992;90(2,I PG-238-244):238–44.
10. Escartin L, Samper MP, Santabarbara J, Labayen I, Alvarez ML, Ayerza A, et al. Determinants of birth size in Northeast Spain. J Matern Neonatal Med. 2014;27(7 PG-677-682):677–82.
11. Lopez-Bermejo A, de Zegher F, Diaz-Silva M, Vicente MP, Valls C, Ibanez L. Cord serum visfatin at term birth: maternal smoking unmasks the relation to foetal growth. Clin Endocrinol (Oxf). 2008;68(1 PG-77-81):77–81.
12. Olsen J. Cigarette smoking in pregnancy and fetal growth. Does the type of tobacco play a role? Int J Epidemiol. 1992;21(2 PG-279-84):279–84.
13. Raisanen S, Gissler M, Sankilampi U, Saari J, Kramer MR, Heinonen S. Contribution of socioeconomic status to the risk of small for gestational age infants - a population-based study of 1,390,165 singleton live births in Finland. Int J Equity Health. 2013;12(PG-).
14. Cnattingius S, Forman MR, Berendes HW, Graubard BI, Isotalo L. Effect of age, parity, and smoking on pregnancy outcome: a population-based study. Am J Obstet Gynecol. 1993;168(1 PG-16-21):16–21.
15. Galão AO, Soder SA, Gerhardt M, Faertes TH, Krüger MS, Pereira DF, et al. Efeitos do fumo materno durante a gestação e complicações perinatais Effects of maternal smoking during pregnancy and perinatal complications. Rev HCPA &amp; Fac Med Univ Fed Rio Gd do Sul. 2009;29(3 PG-218-224):218–24.
16. Newnham JP, Patterson L, James I, Reid SE. Effects of maternal cigarette smoking on ultrasonic measurements of fetal growth and on Doppler flow velocity waveforms. EARLY HUM DEV. 1990;24(1 PG-23-36):23–36.
17. Sanchez-Zamorano LM, Tellez-Rojo MM, Hernandez-Avila M. Effect of smoking during pregnancy on anthropometric characteristics at birth. Salud Publica Mex. 2004;46(6 PG-529-533):529–33.
18. Bernstein IM, Plociennik K, Stahle S, Badger GJ, Secker-Walker R. Impact of maternal cigarette smoking on fetal growth and body composition. Am J Obs Gynecol. 2000;183(4 PG-883-886):883–6.
19. Chelchowska M, Ambroszkiewicz J, Gajewska J, Jablonska-Glab E, Maciejewski TM, Oltarzewski M. Hepcidin and iron metabolism in pregnancy: correlation with smoking and birth weight and length. Biol Trace Elem Res. 2016;173(1 PG-14-20):14–20.
20. Chiavaroli V, Castorani V, Guidone P, Derraik JGB, Liberati M, Chiarelli F, et al. Incidence of infants born small- and large-for-gestational-age in an Italian cohort over a 20-year period and associated risk factors. Ital J Pediatr. 2016;42(42 PG-(26 April 2016)):(26 April 2016).
21. Erickson AC, Arbour LT. Heavy smoking during pregnancy as a marker for other risk factors of adverse birth outcomes: a population-based study in British Columbia, Canada. BMC Public Health. 2012;12(102 PG-(6 February 2012)):(6 February 2012).
22. Fenercioglu AK, Karatekin G, Nuhoglu A. Fetal malnutrition in infants of smokers and passive smokers assessed by clinical assessment of nutritional status scoring. Turkish J Med Sci. 2009;39(6 PG-849-855):849–55.
23. Goncalves-Silva RM V, Valente JG, Ferreira MG, Sichieri R. Household smoking and malnutrition in infants Tabagismo no domicilio e desnutricao em lactentes. Rev Nutr. 2009;22(1 PG-19-27):19–27.
24. Gonçalves-Silva RM, Valente JG, Lemos-Santos MG, Sichieri R. Household smoking and stunting for children under five years. Cad Saude Publica. 2005;21(5 PG-1540-1549):1540–9.
25. Ohmi H, Hirooka K, Mochizuki Y. Fetal growth and the timing of exposure to maternal smoking. Pediatr Int. 2002;44(1 PG-55-59):55–9.
26. Ortega-Garcia JA, Gutierrez-Churango JE, Sanchez-Sauco MF, Martinez-Aroca M, Delgado-Marin JL, Sanchez-Solis M, et al. Head circumference at birth and exposure to tobacco, alcohol and illegal drugs during early pregnancy. Childs Nerv Syst. 2012;28(3 PG-433-439):433–9.
27. Spinillo A, Capuzzo E, Nicola SE, Colonna L, Egbe TO, Zara C. Factors potentiating the smoking-related risk of fetal growth retardation. BR J Obs GYNAECOL. 1994;101(11 PG-954-958):954–8.
28. Backe B. Maternal smoking and age. Effect on birthweight and risk for small-for-gestational age births. Acta Obstet Gynecol Scand. 1993;72(3 PG-172-176):172–6.
29. Bolat F, Eren O, Bolat G, Can E, Comert S, Uslu HS, et al. Maternal smoking during pregnancy and effects on neonatal anthropometry: a prospective study. Turkish J Med Sci. 2012;42(6 PG-999-1005):999–1005.
30. Cnattingius S. Maternal age modifies the effect of maternal smoking on intrauterine growth retardation but not on late fetal death and placental abruption. AM J EPIDEMIOL. 1997;145(4 PG-319-323):319–23.
31. Goldenberg RL, Davis RO, Cliver CP, Cutter GR, Hoffman HJ, Dubard MB, et al. Maternal risk factors and their influence on fetal anthropometric measurements. AM J Obs GYNECOL. 1993;168(4 PG-1197-1205):1197–205.
32. Jaddoe VW V, Verburg BO, de Ridder MAJ, Hofman A, Mackenbach JP, Moll HA, et al. Maternal smoking and fetal growth characteristics in different periods of pregnancy. Am J Epidemiol. 2007;165(10 PG-1207-1215):1207–15.
33. Kuja-Halkola R, D’Onofrio BM, Larsson H, Lichtenstein P. Maternal smoking during pregnancy and adverse outcomes in offspring: Genetic and environmental sources of covariance. Behav Genet. 2014;44(5 PG-456-467):456–67.
34. Luke B, Hawkins MM, Petrie RH. Influence of smoking, weight gain, and pregravid weight for height on intrauterine growth. AM J CLIN NUTR. 1981;34(7 PG-1410-1417):1410–7.
35. Naeye RL. Influence of maternal cigarette smoking during pregnancy on fetal and childhood growth. Obs GYNECOL. 1981;57(1 PG-18-21):18–21.
36. Sasaki S, Kondo T, Sata F, Saijo Y, Katoh S, Nakajima S, et al. Maternal smoking during pregnancy and genetic polymorphisms in the Ah receptor, CYP1A1 and GSTM1 affect infant birth size in Japanese subjects. Mol Hum Reprod. 2006;12(2 PG-77-83):77–83.
37. Spinillo A, Capuzzo E, Nicola S, Colonna L, Iasci A, Zara C. Interaction between fetal gender and risk factors for fetal growth retardation. Am J Obstet Gynecol. 1994;171(5 PG-1273-1277):1273–7.
38. Wen SW, Goldenberg RL, Cutter GR, Hoffman HJ, Cliver SP. Intrauterine growth retardation and preterm delivery: Prenatal risk factors in an indigent population. AM J Obs GYNECOL. 1990;162(1 PG-213-218):213–8.
39. Biosca Pàmies M, Rodríguez Martínez G, Samper Villagrasa MP, Odriozola Grijalba M, Cuadrón Andrés L, Álvarez Sauras ML, et al. Perinatal aspects, growth and feeding of infants born small for gestational age. An Pediatr. 2013;78(1 PG-14-20):14–20.
40. Doménech Martínez E, Fuster Jorge P, Léon Quintana C, Cortabarría Bayona C, Castro Conde JR, Méndez Pérez A. Neonatal morbidity and mortality according to intrauterine growth pattern. An Pediatr. 2005;63(4 PG-300-306):300–6.
41. Hody NA, Stark MJ, Scheil W, Grzeskowiak LE, Clifton VL. Perinatal outcomes following maternal asthma and cigarette smoking during pregnancy. Eur Respir J. 2014;43(3 PG-704-706):704–6.
42. Källén K. Maternal smoking during pregnancy and infant head circumference at birth. Early Hum Dev. 2000;58(3 PG-197-204):197–204.
43. Nieto A, Matorras R, Serra M, Valenzuela P, Molero J. Multivariate analysis of determinants of fetal growth retardation. Eur J Obstet Gynecol Reprod Biol. 1994;53(2 PG-107-13):107–13.
44. Rocio Berlanga M del, Salazar G, Garcia C, Hernandez J. Maternal smoking effects on infant growth. Food Nutr Bull. 2002;23(3, NaN-142–145):142–5.
45. Clausson B, Cnattingius S, Axelsson O. Preterm and term births of small for gestational age infants: A population-based study of risk factors among nulliparous women. Br J Obs Gynaecol. 1998;105(9 PG-1011-1017):1011–7.
46. Cornelius MD, Taylor PM, Geva D, Day NL. Prenatal tobacco and marijuana use among adolescents: effects on offspring gestational age, growth, and morphology. Pediatrics. 1995;95(5 PG-738-743):738–43.
47. Fox NL, Sexton M, Hebel JR. Prenatal exposure to tobacco: I. Effects on physical growth at age three. Int J Epidemiol. 1990;19(1 PG-66-71):66–71.
48. Lampl M, Kuzawa CW, Jeanty P. Prenatal smoke exposure alters growth in limb proportions and head shape in the midgestation human fetus. Am J Hum Biol. 2003;15(4 PG-533-546):533–46.
49. McCowan LME, Thompson J, Taylor RS, Baker P, Dekker G, Roberts C, et al. Prediction of small for gestational age infants using clinical risk factors, biomarkers and ultrasound data: Findings from the scope study. J Paediatr Child Heal. 2014;50(PG-21):21.
50. Berg G van den, Eijsden M van, Galindo-Garre F, Vrijkotte TGM, Gemke RJBJ. Smoking overrules many other risk factors for small for gestational age birth in less educated mothers. Early Hum Dev. 2013;89(7 PG-497-501):497–501.
51. Delgado Pena YP, Rodriguez Martinez G, Samper Villagrasa MP, Caballero Perez V, Cuadron Andres L, Alvarez Sauras ML, et al. Socio-cultural, obstetric and anthropometric characteristics of newborn children of mothers who smoke in Spain Caracteristicas socioculturales, obstetricas y antropometricas de los recien nacidos hijos de madre fumadora. An Pediatr. 2012;76(1 PG-4-9):4–9.
52. Godel JC, Pabst HR, Hodges PE, Johnson KE, Froese GJ, Joffres MR. Smoking and caffeine and alcohol intake during pregnancy in a northern population: Effect on fetal growth. CAN MED ASSOC J. 1992;147(2 PG-181-188):181–8.
53. Hammoud AO, Bujold E, Sorokin Y, Schild C, Krapp M, Baumann P, et al. Smoking in pregnancy revisited: Findings from a large population-based study. Am J Obs Gynecol. 2005;192(6 PG-1856-1863):1856–63.
54. Hanke W, Kalinka J, Sobala W. Sociodemographic and environmental risk factors of preterm delivery and small-for-gestational-age babies. Med Sci Monit. 1998;4(3 PG-505-512):505–12.
55. Krentz H, Voigt M, Hesse V, Guthmann F, Wittwer-Backofen U, Straube S. Somatic Classifications of Neonates Based on Weight-for-Length and Rohrer’s Ponderal Index: Effects of Maternal BMI and Smoking. Geburtshilfe Frauenheilkd. 2011;71(11 PG-973-978):973–8.
56. Lelong N, Blondel B, Kaminski M. Smoking during pregnancy in France between 1972 to 2003: Results from the national perinatal surveys. J Gynecol Obstet Biol La Reprod. 2011;40(1 PG-42-49):42–9.
57. Tenovuo AH, Kero PO, Korvenranta HJ, Erkkola RU, Klemi PJ, Tuominen J. Risk factors associated with severely small for gestational age neonates. AM J PERINATOL. 1988;5(3 PG-267-271):267–71.
58. Król MK, Florek E, Piekoszewski W, Bokiniec R, Kornacka MK. The impact of intrauterine tobacco exposure on the cerebral mass of the neonate based on the measurement of head circumference. Brain Behav. 2012;2(3 PG-243-248):243–8.
59. Lindsay CA, Thomas AJ, Catalano PM. The effect of smoking tobacco on neonatal body composition. Am J Obstet Gynecol. 1997;177(5 PG-1124-8):1124–8.
60. Miller HC, Jekel JF. The epidemiology of white full-term infants with short crown-heel length for gestational ages at birth. YALE J BIOL MED. 1989;62(1 PG-1-12):1–12.
61. Mutlu FS, Ayranci U, Ozdamar K, Yazici S. The effects of maternal cigarette smoking on infant anthropometric measurements. Iran J Public Health. 2008;37(4 PG-65-75):65–75.
62. Rasmussen S, Irgens LM. The effects of smoking and hypertensive disorders on fetal growth. BMC Pregnancy Childbirth. 2006;6(PG-).
63. Xie CB, Epstein LH, Eiden RD, Shenassa ED, Li XH, Liao Y, et al. Stunting at 5 Years Among SGA Newborns. Pediatrics. 2016;137(2 PG-).
64. Zaren B, Lindmark G, Wibell L, Folling I. The effect of smoking on glucose homeostasis and fetal growth in pregnant women. Ups J Med Sci. 2000;105(1 PG-41-56):41–56.
65. Zhang H, Bracken MB. Tree-based risk factor analysis of preterm delivery and small-for- gestational-age birth. AM J EPIDEMIOL. 1995;141(1 PG-70-78):70–8.
66. Heaman M, Kingston D, Chalmers B, Sauve R, Lee L, Young D. Risk factors for preterm birth and small-for-gestational-age births among Canadian women. Paediatr Perinat Epidemiol. 2013;27(1):54–61.
67. Lynch CM, Kelly R, Stuart B, Treumann A, Conroy R, Regan CL. The role of thromboxane A(2) in the pathogenesis of intrauterine growth restriction associated with maternal smoking in pregnancy. Prostaglandins Other Lipid Mediat. 2011;95(1–4):63–7.
68. Xaverius PK, Salas J, Woolfolk CL, Leung F, Yuan J, Chang JJ. Predictors of size for gestational age in St. Louis City and County. Biomed Res Int. 2014;2014(Article ID 515827):8 pages.
69. Samper MP, Jiménez-Muro A, Nerín I, Marqueta A, Ventura P, Rodríguez G. Maternal active smoking and newborn body composition. Early Hum Dev. 2011;88(3):141–5.
70. Espy KA, Fang H, Johnson C, Stopp C, Wiebe SA. Prenatal tobacco exposure: developmental outcomes in the neonatal period. Dev Psychol. 2011;47(1):153–6.
71. Burns L, Mattick RP, Wallace C. Smoking patterns and outcomes in a population of pregnant women with other substance use disorders. Nicotine Tob Res. 2008;10(6):969–74.
72. Hinkle SN, Albert PS, Mendola P, Sjaarda LA, Boghossian NS, Yeung E, et al. Differences in risk factors for incident and recurrent small-for-gestational-age birthweight: a hospital-based cohort study. BJOG. 2014;121(9):1080–8; discussion 1089.
73. Almeida ND, Koren G, Platt RW, Kramer MS. Hair biomarkers as measures of maternal tobacco smoke exposure and predictors of fetal growth. Nicotine Tob Res. 2011;13(5):328–35.
74. Harrod CS, Fingerlin TE, Chasan-Taber L, Reynolds RM, Glueck DH, Dabelea D. Exposure to prenatal smoking and early-life body composition: the healthy start study. Obes (Silver Spring). 2015;23(1):234–41.
75. Conde A, Figueiredo B, Tendais I, Teixeira C, Costa R, Pacheco A, et al. Mother’s anxiety and depression and associated risk factors during early pregnancy: effects on fetal growth and activity at 20-22 weeks of gestation. J Psychosom Obs Gynaecol. 2010;31(2):70–82.
76. Varvarigou AA, Fouzas S, Beratis NG. Effect of prenatal tobacco smoke exposure on fetal growth potential. J Perinat Med. 2010;38(6):683–7.
77. Pavic I, Dodig S, Jurkovic M, Krmek T, Spanovic D. The influence of mother’s active smoking during pregnancy on body mass index of newborns. Coll Antropol. 2011;35(4):1149–54.
78. Pringle PJ, Geary MPP, Rodeck CH, Kingdom JCP, Kayamba-Kay’s S, Hindmarsh PC. The influence of cigarette smoking on antenatal growth, birth size, and the insulin-like growth factor axis. J Clin Endocrinol Metab. 2005 May;90(5):2556–62.
79. Cliver SP, Goldenberg RL, Cutter GR, Hoffman HJ, Davis RO, Nelson KG. The effect of cigarette smoking on neonatal anthropometric measurements. Obstet Gynecol. 1995 Apr;85(4):625–30.
80. Nordentoft M, HC L, Hansen D, Nim J, Pryds O, Rubin P, et al. Intrauterine growth retardation and premature delivery: the influence of maternal smoking and psychosocial factors. Am J Public Health. 1996 Mar;86(3):347–54.
81. Ikeda N, Irie Y, Shibuya K. Determinants of reduced child stunting in Cambodia: analysis of pooled data from three Demographic and Health Surveys. Bull World Health Organ. 2013 May;91(5):341–9.
82. Akahoshi E, Arima K, Miura K, Nishimura T, Abe Y, Yamamoto N, et al. Association of maternal pre-pregnancy weight, weight gain during pregnancy, and smoking with small-for-gestational-age infants in Japan. Early Hum Dev. 2016 Jan;92:33–6.
83. Gao W, Paterson J, Carter S, Percival T. Risk factors for preterm and small-for-gestational-age babies: a cohort from the Pacific Islands Families Study. J Paediatr Child Heal. 2006 Dec;42(12):785–92.
84. Victora CG, Villar J, Barros FC, Ismail LC, Chumlea C, Papageorghiou AT, et al. Anthropometric Characterization of Impaired Fetal Growth: Risk Factors for and Prognosis of Newborns With Stunting or Wasting. JAMA Pediatr. 2015 Jul 6;169(7):e151431–e151431.
85. Rodrigues T, Barros H. Comparison of risk factors for small-for-gestational-age and preterm in a Portuguese cohort of newborns. Matern Child Heal J. 2007 Sep;11(5):417–24.
86. Matijasevich A, MJ B, AM M, AJ B, IS S, FC B. Maternal smoking during pregnancy and offspring growth in childhood: 1993 and 2004 Pelotas cohort studies. Arch Dis Child. 2011 Jun;96(6):519–25.
87. Ganer Herman H, Miremberg H, Nini N, Feit H, Schreiber L, Bar J, et al. The effects of maternal smoking on pregnancy outcome and placental histopathology lesions. Reprod Toxicol. 2016;65:24–8.
88. Kobayashi S, Sata F, Sasaki S, Braimoh TS, Araki A, Miyashita C, et al. Combined effects of AHR, CYP1A1, and XRCC1 genotypes and prenatal maternal smoking on infant birth size: Biomarker assessment in the Hokkaido Study. Reprod Toxicol. 2016;65:295–306.
89. Ounsted M, Moar VA, Scott A. Risk factors associated with small-for-dates and large-for-dates infants. Br J Obstet Gynaecol. 1985 Mar;92(3):226–32.
90. Beaulac-Baillargeon L, Desrosiers C. Caffeine-cigarette interaction on fetal growth. Am J Obstet Gynecol. 1987 Nov;157(5):1236–40.
91. De Scrilli A, Boracchi P, Pardi G, Bevilacqua G, Pezzani FM, Marconi A, et al. Cigarette smoking in pregnancy: relationship to perinatal outcomes in six Italian centres. Genus. 1986;42(1–2):37–52.
92. Day N, Cornelius M, Goldschmidt L, Richardson G, Robles N, Taylor P. The effects of prenatal tobacco and marijuana use on offspring growth from birth through 3 years of age. Neurotoxicol Teratol. 1992;14(6):407–14.
93. Bakketeig LS, Jacobsen G, Hoffman HJ, Lindmark G, Bergsjo P, Molne K, et al. Pre-pregnancy risk factors of small-for-gestational age births among parous women in Scandinavia. Acta Obstet Gynecol Scand. 1993 May;72(4):273–9.
94. Lang JM, Cohen A, Lieberman E. Risk factors for small-for-gestational-age birth in a preterm population. Am J Obstet Gynecol. 1992 May;166(5):1374–8.
95. Jacobson JL, Jacobson SW, Sokol RJ. Effects of prenatal exposure to alcohol, smoking, and illicit drugs on postpartum somatic growth. Alcohol Clin Exp Res. 1994 Apr;18(2):317–23.
96. Arbuckle TE, Sherman GJ. Comparison of the risk factors for pre-term delivery and intrauterine growth retardation. Paediatr Perinat Epidemiol. 1989 Apr;3(2):115–29.
97. Callan NA, Witter FR. Intrauterine growth retardation: characteristics, risk factors and gestational age. Int J Gynaecol Obstet. 1990 Nov;33(3):215–20.
98. Verkerk PH, Buitendijk SE, Verloove-Vanhorick SP. Differential misclassification of alcohol and cigarette consumption by pregnancy outcome. Int J Epidemiol. 1994 Dec;23(6):1218–25.
99. Haste FM, Anderson HR, Brooke OG, Bland JM, Peacock JL. The effects of smoking and drinking on the anthropometric measurements of neonates. Paediatr Perinat Epidemiol. 1991 Jan;5(1):83–92.
100. Spinillo A, Capuzzo E, Piazzi G, Nicola S, Colonna L, Iasci A. Maternal high-risk factors and severity of growth deficit in small for gestational age infants. Early Hum Dev. 1994 Jul;38(1):35–43.
101. Read AW, Stanley FJ. Small-for-gestational-age term birth: the contribution of socio-economic, behavioural and biological factors to recurrence. Paediatr Perinat Epidemiol. 1993 Apr;7(2):177–94.
102. Wen SW, Goldenberg RL, Cutter GR, Hoffman HJ, Cliver SP, Davis RO, et al. Smoking, maternal age, fetal growth, and gestational age at delivery. Am J Obstet Gynecol. 1990 Jan;162(1):53–8.
103. Muscati SK, Gray-Donald K, Newson EE. Interaction of smoking and maternal weight status in influencing infant size. Can J Public Health. 1994;85(6):407–12.
104. Castro LC, Azen C, Hobel CJ, Platt LD. Maternal tobacco use and substance abuse: reported prevalence rates and associations with the delivery of small for gestational age neonates. Obstet Gynecol. 1993 Mar;81(3):396–401.
105. Roquer JM, Figueras J, Botet F, Jimenez R. Influence on fetal growth of exposure to tobacco smoke during pregnancy. Acta Paediatr. 1995 Feb;84(2):118–21.
106. Miyao M, Furuta M, Matsushita Y, Ogiso M, Ishihara S, Teo PC. A matched-pair longitudinal study on the relationship between maternal smoking and head circumference of newborns. Tohoku J Exp Med. 1995 Feb;175(2):135–7.
107. Cnattingius S, Mills JL, Yuen J, Eriksson O, Salonen H. The paradoxical effect of smoking in preeclamptic pregnancies: smoking reduces the incidence but increases the rates of perinatal mortality, abruptio placentae, and intrauterine growth restriction. Am J Obstet Gynecol. 1997 Jul;177(1):156–61.
108. Millar WJ, Chen J. Maternal education and risk factors for small-for-gestational-age births. Heal reports. 1998;10(2):43–51 (Eng); 47–56 (Fre).
109. Perkins SL, Belcher JM, Livesey JF. A Canadian tertiary care centre study of maternal and umbilical cord cotinine levels as markers of smoking during pregnancy: relationship to neonatal effects. Can J Public Health. 1997;88(4):232–7.
110. Wu T, Buck G, Mendola P. Can regular multivitamin/mineral supplementation modify the relation between maternal smoking and select adverse birth outcomes? Ann Epidemiol. 1998 Apr;8(3):175–83.
111. Sprauve ME, Lindsay MK, Drews-Botsch CD, Graves W. Racial patterns in the effects of tobacco use on fetal growth. Am J Obstet Gynecol. 1999 Jul;181(1):S22-7.
112. Wang X, Tager IB, Van Vunakis H, Speizer FE, Hanrahan JP. Maternal smoking during pregnancy, urine cotinine concentrations, and birth outcomes. A prospective cohort study. Int J Epidemiol. 1997 Oct;26(5):978–88.
113. Eliopoulos C, Klein J, Chitayat D, Greenwald M, Koren G. Nicotine and cotinine in maternal and neonatal hair as markers of gestational smoking. Clin Invest Med. 1996 Aug;19(4):231–42.
114. Muscati SK, Koski KG, Gray-Donald K. Increased energy intake in pregnant smokers does not prevent human fetal growth retardation. J Nutr. 1996 Dec;126(12):2984–9.
115. Zaren B, Lindmark G, Gebre-Medhin M. Maternal smoking and body composition of the newborn. Acta Paediatr. 1996 Feb;85(2):213–9.
116. Horta BL, Victora CG, Menezes AM, Halpern R, Barros FC. Low birthweight, preterm births and intrauterine growth retardation in relation to maternal smoking. Paediatr Perinat Epidemiol. 1997 Apr;11(2):140–51.
117. Schramm WF. Smoking during pregnancy: Missouri longitudinal study. Paediatr Perinat Epidemiol. 1997 Jan;11 Suppl 1:73–83.
118. Cnattingius S, Haglund B. Decreasing smoking prevalence during pregnancy in Sweden: the effect on small-for-gestational-age births. Am J Public Health. 1997 Mar;87(3):410–3.
119. Dejin-Karlsson E, Ostergren P-O. Psychosocial factors, lifestyle, and fetal growth: the added value of both pre- and post-natal assessments. Eur J Public Health. 2003 Sep;13(3):210–7.
120. Karatza AA, Varvarigou A, Beratis NG. Growth up to 2 years in relationship to maternal smoking during pregnancy. Clin Pediatr (Phila). 2003;42(6):533–41.
121. Lindley AA, Becker S, Gray RH, Herman AA. Effect of continuing or stopping smoking during pregnancy on infant birth weight, crown-heel length, head circumference, ponderal index, and brain:body weight ratio. Am J Epidemiol. 2000 Aug;152(3):219–25.
122. Kirchengast S, Hartmann B. Nicotine consumption before and during pregnancy affects not only newborn size but also birth modus. J Biosoc Sci. 2003 Apr;35(2):175–88.
123. Mitchell EA, Thompson JMD, Robinson E, Wild CJ, Becroft DMO, Clark PM, et al. Smoking, nicotine and tar and risk of small for gestational age babies. Acta Paediatr. 2002;91(3):323–8.
124. Thompson JM, Clark PM, Robinson E, Becroft DM, Pattison NS, Glavish N, et al. Risk factors for small-for-gestational-age babies: The Auckland Birthweight Collaborative Study. J Paediatr Child Health. 2001 Aug;37(4):369–75.
125. Zambonato AMK, Pinheiro RT, Horta BL, Tomasi E. [Risk factors for small-for-gestational age births among infants in Brazil]. Rev Saude Publica. 2004 Feb;38(1):24–9.
126. Dejmek J, Solansk y I, Podrazilova K, Sram RJ. The exposure of nonsmoking and smoking mothers to environmental tobacco smoke during different gestational phases and fetal growth. Environ Health Perspect. 2002 Jun;110(6):601–6.
127. Zaren B, Lindmark G, Bakketeig L. Maternal smoking affects fetal growth more in the male fetus. Paediatr Perinat Epidemiol. 2000 Apr;14(2):118–26.
128. Kallen K. The impact of maternal smoking during pregnancy on delivery outcome. Eur J Public Health. 2001 Sep;11(3):329–33.
129. Zeitlin JA, Ancel PY, Saurel-Cubizolles MJ, Papiernik E. Are risk factors the same for small for gestational age versus other preterm births? Am J Obstet Gynecol. 2001 Jul;185(1):208–15.
130. Pichini S, Puig C, Garcia-Algar O O, Pacifici R, Figueroa C, Vall O, et al. [Neonatal effects of smoking habit during pregnancy and sociodemographic determinants in Barcelona, Spain]. Med Clin (Barc). 2002 Jan;118(2):53–6.
131. Kanellopoulos TA, Varvarigou AA, Karatza AA, Beratis NG. Course of growth during the first 6 years in children exposed in utero to tobacco smoke. Eur J Pediatr. 2007 Jul;166(7):685–92.
132. Ingvarsson RF, Bjarnason AO, Dagbjartsson A, Hardardottir H, Haraldsson A, Thorkelsson T. The effects of smoking in pregnancy on factors influencing fetal growth. Acta Paediatr. 2007 Mar;96(3):383–6.
133. Infante-Rivard C, Weinberg CR, Guiguet M. Xenobiotic-metabolizing genes and small-for-gestational-age births: interaction with maternal smoking. Epidemiology. 2006 Jan;17(1):38–46.
134. Schwendemann WD, O’Brien JM, Barton JR, Milligan DA, Istwan N. Modifiable risk factors for growth restriction in twin pregnancies. Am J Obstet Gynecol. 2005 May;192(5):1440–2.
135. Raatikainen K, Huurinainen P, Heinonen S. Smoking in early gestation or through pregnancy: a decision crucial to pregnancy outcome. Prev Med (Baltim). 2007 Jan;44(1):59–63.
136. Kleijer ME, Dekker GA, Heard AR. Risk factors for intrauterine growth restriction in a socio-economically disadvantaged region. J Matern Fetal Neonatal Med. 2005 Jul;18(1):23–30.
137. Infante-Rivard C. Caffeine intake and small-for-gestational-age birth: modifying effects of xenobiotic-metabolising genes and smoking. Paediatr Perinat Epidemiol. 2007 Jul;21(4):300–9.
138. Okah FA, Hoff GL, Dew P, Cai J. Cumulative and residual risks of small for gestational age neonates after changing pregnancy-smoking behaviors. Am J Perinatol. 2007 Mar;24(3):191–6.
139. Salihu HM, Shumpert MN, Aliyu MH, Kirby RS, Alexander GR. Smoking-associated fetal morbidity among older gravidas: a population study. Acta Obstet Gynecol Scand. 2005 Apr;84(4):329–34.
140. Panaretto K, Lee H, Mitchell M, Larkins S, Manessis V, Buettner P, et al. Risk factors for preterm, low birth weight and small for gestational age birth in urban Aboriginal and Torres Strait Islander women in Townsville. Aust N Z J Public Health. 2006 Apr;30(2):163–70.
141. Salihu HM, Aliyu MH, Kirby RS. In utero nicotine exposure and fetal growth inhibition among twins. Am J Perinatol. 2005 Nov;22(8):421–7.
142. England LJ, Grauman A, Qian C, Wilkins DG, Schisterman EF, Yu KF, et al. Misclassification of maternal smoking status and its effects on an epidemiologic study of pregnancy outcomes. Nicotine Tob Res. 2007 Oct;9(10):1005–13.
143. Lanting CI, Buitendijk SE, Crone MR, Segaar D, Bennebroek Gravenhorst J, van Wouwe JP. Clustering of socioeconomic, behavioural, and neonatal risk factors for infant health in pregnant smokers. PLoS One. 2009;4(12):e8363.
144. Varvarigou AA, Asimakopoulou A, Beratis NG. Impact of maternal smoking on birth size: effect of parity and sex dimorphism. Neonatology. 2009;95(1):61–7.
145. Okah FA, Cai J, Dew PC, Hoff GL. Risk factors for recurrent small-for-gestational-age birth. Am J Perinatol. 2010 Jan;27(1):1–7.
146. Aagaard-Tillery K, Spong CY, Thom E, Sibai B, Wendel GJ, Wenstrom K, et al. Pharmacogenomics of maternal tobacco use: metabolic gene polymorphisms and risk of adverse pregnancy outcomes. Obstet Gynecol. 2010 Mar;115(3):568–77.
147. McCowan LME, Dekker GA, Chan E, Stewart A, Chappell LC, Hunter M, et al. Spontaneous preterm birth and small for gestational age infants in women who stop smoking early in pregnancy: prospective cohort study. BMJ. 2009;338:b1081.
148. Gray TR, Magri R, Shakleya DM, Huestis MA. Meconium nicotine and metabolites by liquid chromatography-tandem mass spectrometry: differentiation of passive and nonexposure and correlation with neonatal outcome measures. Clin Chem. 2008 Dec;54(12):2018–27.
149. Aagaard-Tillery KM, Porter TF, Lane RH, Varner MW, Lacoursiere DY. In utero tobacco exposure is associated with modified effects of maternal factors on fetal growth. Am J Obstet Gynecol. 2008 Jan;198(1):66.e1-6.
150. Delpisheh A, Brabin L, Drummond S, Brabin BJ. Prenatal smoking exposure and asymmetric fetal growth restriction. Ann Hum Biol. 2008;35(6):573–83.
151. Delpisheh A, Brabin L, Topping J, Reyad M, Tang A-W, Brabin BJ. A case-control study of CYP1A1, GSTT1 and GSTM1 gene polymorphisms, pregnancy smoking and fetal growth restriction. Eur J Obstet Gynecol Reprod Biol. 2009 Mar;143(1):38–42.
152. Fitzgerald K, Cai J, Hoff G, Dew P, Okah F. Clinical manifestation of small-for-gestational-age risk pregnancy from smoking is gestational age dependent. Am J Perinatol. 2007 Oct;24(9):519–24.
153. Sasaki S, Sata F, Katoh S, Saijo Y, Nakajima S, Washino N, et al. Adverse birth outcomes associated with maternal smoking and polymorphisms in the N-Nitrosamine-metabolizing enzyme genes NQO1 and CYP2E1. Am J Epidemiol. 2008 Mar;167(6):719–26.
154. Figueras F, Meler E, Eixarch E, Francis A, Coll O, Gratacos E, et al. Association of smoking during pregnancy and fetal growth restriction: subgroups of higher susceptibility. Eur J Obstet Gynecol Reprod Biol. 2008 Jun;138(2):171–5.
155. Ekblad M, Korkeila J, Parkkola R, Lapinleimu H, Haataja L, Lehtonen L. Maternal smoking during pregnancy and regional brain volumes in preterm infants. J Pediatr. 2010 Feb;156(2):185–90.e1.
156. Andersen MR, Simonsen U, Uldbjerg N, Aalkjaer C, Stender S. Smoking cessation early in pregnancy and birth weight, length, head circumference, and endothelial nitric oxide synthase activity in umbilical and chorionic vessels: an observational study of healthy singleton pregnancies. Circulation. 2009 Feb;119(6):857–64.
157. Polakowski LL, Akinbami LJ, Mendola P. Prenatal smoking cessation and the risk of delivering preterm and small-for-gestational-age newborns. Obstet Gynecol. 2009 Aug;114(2 Pt 1):318–25.
158. Quinton AE, Cook C-M, Peek MJ. The relationship between cigarette smoking, endothelial function and intrauterine growth restriction in human pregnancy. BJOG. 2008 May;115(6):780–4.
159. Tsukamoto H, Fukuoka H, Koyasu M, Nagai Y, Takimoto H. Risk factors for small for gestational age. Pediatr Int. 2007 Dec;49(6):985–90.
160. Fenercioglu AK, Tamer I, Karatekin G, Nuhoglu A. Impaired postnatal growth of infants prenatally exposed to cigarette smoking. Tohoku J Exp Med. 2009 Jul;218(3):221–8.
161. Voigt M, Briese V, Jorch G, Henrich W, Schneider KTM, Straube S. The influence of smoking during pregnancy on fetal growth. Considering daily cigarette consumption and the SGA rate according to length of gestation. Z Geburtshilfe Neonatol. 2009 Oct;213(5):194–200.
162. Meyer S, Raisig A, Gortner L, Ong MF, Bucheler M, Tutdibi E. In utero tobacco exposure: the effects of heavy and very heavy smoking on the rate of SGA infants in the Federal State of Saarland, Germany. Eur J Obstet Gynecol Reprod Biol. 2009 Sep;146(1):37–40.
163. Leviton A, Kuban K, Allred EN, Hecht JL, Onderdonk A, O’Shea TM, et al. Antenatal antecedents of a small head circumference at age 24-months post-term equivalent in a sample of infants born before the 28th post-menstrual week. Early Hum Dev. 2010 Aug;86(8):515–21.
164. Hindmarsh PC, Geary MPP, Rodeck CH, Kingdom JCP, Cole TJ. Factors predicting ante- and postnatal growth. Pediatr Res. 2008 Jan;63(1):99–102.
165. Inde Y, Satomi M, Iwasaki N, Ono S, Yamashita E, Igarashi M, et al. Maternal risk factors for small-for-gestational age newborns in Japanese dichorionic twins. J Obstet Gynaecol Res. 2011 Jan;37(1):24–31.
166. Watanabe H, Inoue K, Doi M, Matsumoto M, Ogasawara K, Fukuoka H, et al. Risk factors for term small for gestational age infants in women with low prepregnancy body mass index. J Obstet Gynaecol Res. 2010 Jun;36(3):506–12.
167. Titova OE, Ayvazova EA, Bichkaeva FA, Brooks SJ, Chumakova GN, Schioth HB, et al. The influence of active and passive smoking during pregnancy on umbilical cord blood levels of vitamins A and E and neonatal anthropometric indices. Br J Nutr. 2012 Oct;108(8):1341–5.
168. Krstev S, Marinkovic J, Simic S, Kocev N, Bondy SJ. The influence of maternal smoking and exposure to residential ETS on pregnancy outcomes: a retrospective national study. Matern Child Health J. 2013 Nov;17(9):1591–8.
169. Quesada O, Gotman N, Howell HB, Funai EF, Rounsaville BJ, Yonkers KA. Prenatal hazardous substance use and adverse birth outcomes. J Matern Fetal Neonatal Med. 2012 Aug;25(8):1222–7.
170. Durmus B, Kruithof CJ, Gillman MH, Willemsen SP, Hofman A, Raat H, et al. Parental smoking during pregnancy, early growth, and risk of obesity in preschool children: the Generation R Study. Am J Clin Nutr. 2011 Jul;94(1):164–71.
171. Zhang L, Gonzalez-Chica DA, Cesar JA, Mendoza-Sassi RA, Beskow B, Larentis N, et al. [Maternal smoking during pregnancy and anthropometric measurements of newborns: a population-based study in southern of Brazil]. Cad Saude Publica. 2011 Sep;27(9):1768–76.
172. Wen X, Triche EW, Hogan JW, Shenassa ED, Buka SL. Birth weight and adult hypercholesterolemia: subgroups of small-for-gestational-age based on maternal smoking status during pregnancy. Epidemiology. 2010 Nov;21(6):786–90.
173. Himes SK, Stroud LR, Scheidweiler KB, Niaura RS, Huestis MA. Prenatal tobacco exposure, biomarkers for tobacco in meconium, and neonatal growth outcomes. J Pediatr. 2013 May;162(5):970–5.
174. Voigt M, Jorch G, Briese V, Kwoll G, Borchardt U, Straube S. The combined effect of maternal body mass index and smoking status on perinatal outcomes - an analysis of the german perinatal survey. Z Geburtshilfe Neonatol. 2011 Feb;215(1):23–8.
175. Prabhu N, Smith N, Campbell D, Craig LC, Seaton A, Helms PJ, et al. First trimester maternal tobacco smoking habits and fetal growth. Thorax. 2010 Mar;65(3):235–40.
176. Goetzinger KR, Cahill AG, Macones GA, Odibo AO. The relationship between maternal body mass index and tobacco use on small-for-gestational-age infants. Am J Perinatol. 2012 Mar;29(3):153–8.
177. Kahn SR, Almeida ND, McNamara H, Koren G, Genest JJ, Dahhou M, et al. Smoking in preeclamptic women is associated with higher birthweight for gestational age and lower soluble fms-like tyrosine kinase-1 levels: a nested case control study. BMC Pregnancy Childbirth. 2011;11:91.
178. Gray TR, Eiden RD, Leonard KE, Connors G, Shisler S, Huestis MA. Nicotine and metabolites in meconium as evidence of maternal cigarette smoking during pregnancy and predictors of neonatal growth deficits. Nicotine Tob Res. 2010 Jun;12(6):658–64.
179. Campbell MK, Cartier S, Xie B, Kouniakis G, Huang W, Han V. Determinants of small for gestational age birth at term. Paediatr Perinat Epidemiol. 2012 Nov;26(6):525–33.
180. McCowan LME, Roberts CT, Dekker GA, Taylor RS, Chan EHY, Kenny LC, et al. Risk factors for small-for-gestational-age infants by customised birthweight centiles: data from an international prospective cohort study. BJOG. 2010 Dec;117(13):1599–607.
181. Baba S, Wikstrom A-K, Stephansson O, Cnattingius S. Changes in snuff and smoking habits in Swedish pregnant women and risk for small for gestational age births. BJOG. 2013 Mar;120(4):456–62.
182. Bickerstaff M, Beckmann M, Gibbons K, Flenady V. Recent cessation of smoking and its effect on pregnancy outcomes. Aust N Z J Obstet Gynaecol. 2012 Feb;52(1):54–8.
183. Mehaffey K, Higginson A, Cowan J, Osborne GM, Arbour LT. Maternal smoking at first prenatal visit as a marker of risk for adverse pregnancy outcomes in the Qikiqtaaluk (Baffin) Region. Rural Remote Health. 2010;10(3):1484.
184. Anderson NH, Sadler LC, Stewart AW, Fyfe EM, McCowan LME. Independent risk factors for infants who are small for gestational age by customised birthweight centiles in a multi-ethnic New Zealand population. Aust N Z J Obstet Gynaecol. 2013 Apr;53(2):136–42.
185. Kayemba-Kay’s S, Ribrault A, Burguet A, Gouyon JB, Riethmuller D, Menget A, et al. Maternal smoking during pregnancy and fetal growth. Effects in preterm infants of gestational age less than 33 weeks. Swiss Med Wkly. 2010;140:w13139.
186. Tveit JVH, Saastad E, Stray-Pedersen B, Bordahl PE, Froen JF. Concerns for decreased foetal movements in uncomplicated pregnancies--increased risk of foetal growth restriction and stillbirth among women being overweight, advanced age or smoking. J Matern Fetal Neonatal Med. 2010 Oct;23(10):1129–35.
187. Miyake Y, Tanaka K, Arakawa M. Active and passive maternal smoking during pregnancy and birth outcomes: the Kyushu Okinawa maternal and child health study. BMC Pregnancy Childbirth. 2013;13:157.
188. Ko T-J, Tsai L-Y, Chu L-C, Yeh S-J, Leung C, Chen C-Y, et al. Parental smoking during pregnancy and its association with low birth weight, small for gestational age, and preterm birth offspring: a birth cohort study. Pediatr Neonatol. 2014 Feb;55(1):20–7.
189. Waldenstrom U, Aasheim V, Nilsen ABV, Rasmussen S, Pettersson HJ, Schytt E. Adverse pregnancy outcomes related to advanced maternal age compared with smoking and being overweight. Obstet Gynecol. 2014 Jan;123(1):104–12.
190. Blatt K, Moore E, Chen A, Van Hook J, DeFranco EA. Association of reported trimester-specific smoking cessation with fetal growth restriction. Obstet Gynecol. 2015 Jun;125(6):1452–9.
191. Seravalli V, Block-Abraham DM, Turan OM, Doyle LE, Blitzer MG, Baschat AA. Second-trimester prediction of delivery of a small-for-gestational-age neonate: integrating sequential Doppler information, fetal biometry, and maternal characteristics. Prenat Diagn. 2014 Nov;34(11):1037–43.
192. Juarez SP, Wagner P, Merlo J. Applying measures of discriminatory accuracy to revisit traditional risk factors for being small for gestational age in Sweden: a national cross-sectional study. BMJ Open. 2014;4(7):e005388.
193. Suzuki K, Sato M, Zheng W, Shinohara R, Yokomichi H, Yamagata Z. Effect of maternal smoking cessation before and during early pregnancy on fetal and childhood growth. J Epidemiol. 2014;24(1):60–6.
194. Walfisch A, Nikolovski S, Talevska B, Hallak M. Fetal growth restriction and maternal smoking in the Macedonian Roma population: a causality dilemma. Arch Gynecol Obstet. 2013 Jun;287(6):1131–6.
195. Hodyl NA, Grzeskowiak LE, Stark MJ, Scheil W, Clifton VL. The impact of Aboriginal status, cigarette smoking and smoking cessation on perinatal outcomes in South Australia. Med J Aust. 2014 Sep;201(5):274–8.
196. Fried PA, O’Connell CM. A comparison of the effects of prenatal exposure to tobacco, alcohol, cannabis and caffeine on birth size and subsequent growth. Neurotoxicol Teratol. 1987;9(2):79–85.
197. Scott A, Moar V, Ounsted M. The relative contributions of different maternal factors in small-for-gestational-age pregnancies. Eur J Obstet Gynecol Reprod Biol. 1981 Sep;12(3):157–65.
198. Bosley AR, Sibert JR, Newcombe RG. Effects of maternal smoking on fetal growth and nutrition. Arch Dis Child. 1981 Sep;56(9):727–9.
199. D’Souza SW, Black P, Richards B. Smoking in pregnancy: associations with skinfold thickness, maternal weight gain, and fetal size at birth. Br Med J (Clin Res Ed). 1981 May;282(6277):1661–3.
200. Scholl TO, Salmon RW, Miller LK. Smoking and adolescent pregnancy outcome. J Adolesc Health Care. 1986 Nov;7(6):390–4.
201. Wertelecki W, Hoff C, Zansky S. Maternal smoking: greater effect on males, fetal tobacco syndrome? Teratology. 1987 Jun;35(3):317–20.
202. Chernick V, Childiaeva R, Ioffe S. Effects of maternal alcohol intake and smoking on neonatal electroencephalogram and anthropometric measurements. Am J Obstet Gynecol. 1983 May;146(1):41–7.
203. Tenovuo A, Kero P, Piekkala P, Korvenranta H, Sillanpää M, Erkkola R. Growth of 519 small for gestational age infants during the first two years of life. Acta Paediatr Scand. 1987 Jul;76(4):636–46.
204. Ong KKL, Preece MA, Emmett PM, Ahmed ML, Dunger DB, ALSPAC Study Team. Size at birth and early childhood growth in relation to maternal smoking, parity and infant breast-feeding: longitudinal birth cohort study and analysis. Pediatr Res. 2002 Dec;52(6):863–7.
205. Vardavas CI, Chatzi L, Patelarou E, Plana E, Sarri K, Kafatos A, et al. Smoking and smoking cessation during early pregnancy and its effect on adverse pregnancy outcomes and fetal growth. Eur J Pediatr. 2010 Jun 2;169(6):741–8.
206. England LJ, Kim SY, Shapiro-Mendoza CK, Wilson HG, Kendrick JS, Satten GA, et al. Maternal smokeless tobacco use in Alaska Native women and singleton infant birth size. Acta Obstet Gynecol Scand. 2012 Jan;91(1):93–103.
207. Jacobson JL, Jacobson SW, Sokol RJ, Martier SS, Ager JW, Shankaran S. Effects of alcohol use, smoking, and illicit drug use on fetal growth in black infants. J Pediatr. 1994;124(5):757–64.
208. Fried PA, Watkinson B, Gray R. Growth from Birth to Early Adolescence in Offspring Prenatally Exposed to Cigarettes and Marijuana. Neurotoxicol Teratol. 1999;21(5):513–25.
209. Vielwerth SE, Jensen RB, Larsen T, Greisen G. The impact of maternal smoking on fetal and infant growth. Early Hum Dev. 2007;83(8):491–5.
210. Shankaran S, Das A, Bauer CR, Bada HS, Lester B, Wright LL, et al. Association between patterns of maternal substance use and infant birth weight, length, and head circumference. Pediatrics. 2004 Aug;114(2):e226-34.
