# Supplementary material for: The association between active tobacco use during pregnancy and growth outcomes of children under five years of age: a systematic review and meta-analysis
Source: BMC Public Health. 2018 Dec 13;18:1372. doi: 10.1186/s12889-018-6137-7 (PMC6293508; doi:10.1186/s12889-018-6137-7)
Supplement: Supplementary file 3 — Table S1. Characteristics of studies included in the systematic review and meta-analysis. Description of data: A complete list of the studies included in the systematic review and meta-analysis. The data included shows each study’s location, design, sample size, growth outcome, and quality of research. (DOCX 35 kb) [file 12889_2018_6137_MOESM3_ESM.docx]

**Additional file 3: Table S1**. Characteristics of studies included in the systematic review and meta-analysis.

| **Study** | **Location** | **Study design** | **Sample size** | | **Exposure data** | **Growth outcome** | **Quality** | **Meta-analysis** |  |
| --- | --- | --- | --- | --- | --- | --- | --- | --- | --- |
|  | **Asia** |  |  | |  |  |  |  |  |
| Ikeda, 2013 | Cambodia | Cross-sectional | 10,366 | | SR | Stunting | Good | No |  |
| Miyao, 1995 | Japan | Case-control | 94 | | SR | HC | Fair | Yes |  |
| Ohmi, 2002 | Japan | Retrospective cohort | 1,194 | | SR | Length | Good | No |  |
| Sasaki, 2006 | Japan | Prospective cohort | 293 | | SR | Length | Fair | Yes |  |
| Tsukamoto, 2007 | Japan | Retrospective cohort | 2,972 | | SR | SGA | Fair | Yes |  |
| Sasaki, 2008 | Japan | Prospective cohort | 460 | | SR | Length, HC | Good | Yes |  |
| Watanabe, 2010 | Japan | Cross-sectional | 3,280 | | SR | SGA | Fair | Yes |  |
| Inde, 2011 | Japan | Retrospective cohort | 680 | | SR | SGA | Good | Yes |  |
| Miyake, 2013 | Japan | Cross-sectional | 1,565 | | SR | SGA | Fair | Yes |  |
| Suzuki, 2014 | Japan | Prospective cohort | 2,230 | | SR | SGA | Fair | Yes |  |
| Akahoshi, 2016 | Japan | Cross-sectional | 621 | | SR | SGA | Fair | Yes |  |
| Kobayashia, 2016 | Japan | Prospective cohort | 3,263 | | SR + biomarker | Length, HC | Good | Yes |  |
| Ko, 2014 | Taiwan | Prospective cohort | 21,248 | | SR | SGA | Fair | Yes |  |
|  | **Europe** |  |  | |  |  |  |  |  |
| Kirchengast, 2003 | Austria | Retrospective cohort | 7,803 | | SR | Length, HC | Fair | Yes |  |
| Lampl, 2003 | Belgium | Cross-sectional | 400 | | SR | Length | Good | Yes |  |
| Pavic, 2011 | Croatia | Retrospective cohort | 219 | | SR | SGA | Fair | Yes |  |
| Dejmek, 2002 | Czech Republic | Retrospective cohort | 6,866 | | SR | SGA | Good | Yes |  |
| Olsen, 1992 | Denmark | Cross-sectional | 10,485 | | SR | Length, HC | Fair | Yes |  |
| Nordentoft, 1996 | Denmark | Cross-sectional | 2,432 | | SR | SGA | Good | Yes |  |
| Vielwerth, 2007 | Denmark | Prospective cohort | 269 | | SR | Length | Fair | No |  |
| Andersen, 2009 | Denmark | Cross-sectional | 266 | | SR + biomarker | Length, HC | Fair | No |  |
| Tenovuo, 1987 | Finland | Prospective cohort | 5,356 | | SR | SGA, Length | Fair | Yes |  |
| Raatikainen, 2007 | Finland | Cross-sectional | 25,591 | | SR | SGA | Fair | Yes |  |
| Ekblad, 2010 | Finland | Prospective cohort | 232 | | SR | HC | Good | Yes |  |
| Raisanen, 2013 | Finland | Case-control | 1,390,165 | | SR | SGA | Good | No |  |
| Kayemba-Kay, 2010 | France | Prospective cohort | 719 | | SR | Length, HC | Fair | Yes |  |
| Lelong, 2011 | France | Retrospective cohort | 56,452 | | SR | Length, HC | Fair | No |  |
| Hammoud, 2005 | Germany | Retrospective cohort | 170,254 | | SR | SGA | Good | No |  |
| Meyer, 2009 | Germany | Cross-sectional | 14,593 | | SR | SGA | Fair | Yes |  |
| Voigt, 2009 | Germany | Unclear | 643,288 | | SR | SGA | Fair | No |  |
| Voigt, 2011 | Germany | Retrospective cohort | 508,926 | | SR | SGA | Fair | No |  |
| Krentz, 2011 | Germany | Retrospective cohort | 433,643 | | SR | SGA | Fair | Yes |  |
| Karatza, 2003 | Greece | Case-control | 408 | | SR | Length, HC | Fair | Yes |  |
| Kanellopoulos, 2007 | Greece | Case-control | 200 | | SR | Length, HC | Good | Yes |  |
| Varvarigou, 2009 | Greece | Prospective cohort | 2,108 | | SR | Length, HC | Fair | Yes |  |
| Varvarigou, 2010 | Greece | Cross-sectional | 3,227 | | SR | SGA | Fair | Yes |  |
| Vardavas, 2010 | Greece | Prospective cohort | 1,400 | | SR | SGA, Length, HC | Poor | Yes |  |
| Ingvarsson, 2007 | Iceland | Case-control | 90 | | SR | Length, HC | Good | Yes |  |
| Herman, 2016 | Israel | Cross-sectional | 1,203 | | SR | SGA | Good | Yes |  |
| Scrilli, 1986 | Italy | Unclear | 36,544 | | SR | Length, HC | Fair | Yes |  |
| Spinillo, 1994a | Italy | Case-control | 1,312 | | SR | SGA | Good | Yes |  |
| Spinillo, 1994b  Spinillo, 1994c | Italy  Italy | Case-control  Case-control | 1,397  1,041 | | SR  SR | SGA  SGA | Fair  Good | Yes  Yes |  |
| Chiavaroli, 2016 | Italy | Retrospective cohort | 5,896 | | SR | SGA | Good | Yes |  |
| Walfisch, 2013 | Macedonia | Retrospective cohort | 2,108 | | SR | SGA, HC | Fair | Yes |  |
| Verkerk, 1994 | Netherlands | Prospective cohort | 2,806 | | SR | SGA | Good | No |  |
| Jaddoe, 2007 | Netherlands | Prospective cohort | 7,098 | | SR | HC | Good | No |  |
| Lanting, 2009 | Netherlands | Cross-sectional | 14,553 | | SR | SGA | Fair | Yes |  |
| Durmus, 2011 | Netherlands | Prospective cohort | 5,342 | | SR | SGA, Length, HC | Fair | No |  |
| Berg, 2013 | Netherlands | Prospective cohort | 3,793 | | SR | SGA | Fair | Yes |  |
| Backe, 1993 | Norway | Prospective cohort | 1,908 | | SR | SGA | Fair | No |  |
| Bakketeig, 1993 | Norway | Unclear | 1,945 | | SR | SGA | Fair | No |  |
| Rasmussen, 2006 | Norway | Cross-sectional | 215,598 | | SR | SGA | Good | Yes |  |
| Tveit, 2010 | Norway | Prospective cohort | 2,374 | | SR | SGA | Good | Yes |  |
| Hanke, 1998 | Poland | Prospective cohort | 987 | | SR | SGA | Poor | No |  |
| Krol, 2012 | Poland | Unclear | 147 | | SR + biomarker | HC | Good | No |  |
| Chelchowska, 2016 | Poland | Prospective cohort | 296 | | SR + biomarker | Length, HC | Good | Yes |  |
| Rodrigues, 2007 | Portugal | Prospective cohort | 4,193 | | SR | SGA | Good | Yes |  |
| Conde, 2010 | Portugal | Prospective cohort | 147 | | SR | Length, HC | Fair | No |  |
| Titova, 2012 | Russia | Prospective cohort | 43 | | SR | Length, HC | Poor | No |  |
| Krstev, 2013 | Serbia | Retrospective cohort | 2,613 | | SR | Length, HC | Fair | No |  |
| Nieto, 1994 | Spain | Case-control | 370 | | SR | SGA | Fair | Yes |  |
| Roquer, 1995 | Spain | Unclear | 129 | | SR | SGA, Length | Fair | Yes |  |
| Pichini, 2002 | Spain | Unclear | 419 | | SR + biomarker | Length, HC | Good | Yes |  |
| Doménech Martínez, 2005 | Spain | Retrospective cohort | 12,311 | | SR | SGA | Good | No |  |
| Figueras, 2008 | Spain | Retrospective cohort | 13,661 | | SR | SGA | Fair | Yes |  |
| Lopez-Bermejo, 2008 | Spain | Cross-sectional | 78 | | SR | Length, HC | Fair | Yes |  |
| Ortega-Garcia, 2011 | Spain | Prospective cohort | 419 | | SR | HC | Good | No |  |
| Iniguez, 2012 | Spain | Unclear | 780 | | SR + biomarker | Length, HC | Good | Yes |  |
| Samper, 2012 | Spain | Unclear | 1,216 | | SR | Length, HC | Good | Yes |  |
| Delgado Peña, 2012 | Spain | Prospective cohort | | 1,499 | SR | SGA, Length, HC | Good | Yes | |
| Escartin, 2013 | Spain | Cross-sectional | | 1,442 | SR | Length | Good | Yes |  |
| Biosca Pàmies, 2013 | Spain | Prospective cohort | 1,596 | | SR | SGA | Fair | No |  |
| Ahlsten, 1993 | Sweden | Prospective cohort | 3,476 | | SR | SGA, Length | Fair | Yes |  |
| Cnattingius, 1993 | Sweden | Prospective cohort | 538,829 | | SR | SGA | Good | No |  |
| Zaren, 1996 | Sweden | Prospective cohort | 874 | | SR + biomarker | SGA, Length, HC | Fair | Yes |  |
| Cnattingius, 1997a | Sweden | Prospective cohort | 1,048,139 | | SR | SGA | Good | No |  |
| Cnattingius, 1997b | Sweden | Retrospective cohort | 1,057,711 | | SR | SGA | Good | No |  |
| Cnattingius, 1997c | Sweden | Unclear | 317,652 | | SR | SGA | Good | No |  |
| Clausson, 1998 | Sweden | Cross-sectional | 96,662 | | SR | SGA | Fair | Yes |  |
| Lindley, 2000 | Sweden | Cross-sectional | 15,185 | | SR | Length, HC | Fair | No |  |
| Zaren, 2000a | Sweden | Prospective cohort | 499 | | SR | Length, HC | Fair | Yes |  |
| Kallen, 2000 | Sweden | Prospective cohort | 1,362,169 | | SR | HC | Fair | No |  |
| Kallen, 2001 | Sweden | Retrospective cohort | 1,413,811 | | SR | HC | Good | No |  |
| England, 2003 | Sweden | Unclear | 23,524 | | SR | SGA | Fair | No |  |
| Dejin-Karlsson, 2003 | Sweden | Prospective cohort | 747 | | SR | SGA | Fair | No |  |
| Baba, 2012* | Sweden | Prospective cohort | 846,411 | | SR | SGA | Good | No |  |
| Juarez, 2014 | Sweden | Cross-sectional | 731,989 | | SR | SGA | Good | No |  |
| Kuja-Halkola, 2014 | Sweden | Case-control | 2,754,626 | | SR + biomarker | SGA | Good | Yes |  |
| Zaren, 2000b | Sweden, Norway | Prospective cohort | 856 | | SR + biomarker | Length, HC | Good | Yes |  |
| Waldenstrom, 2014 | Sweden, Norway | Unclear | 955,804 | | SR | SGA | Good | No |  |
| Mutlu, 2008 | Turkey | Retrospective cohort | 6,332 | | SR | Length, HC | Fair | No |  |
| Kutan Fenergioglu, 2009a | Turkey | Unclear | 266 | | SR | Length, HC | Good | Yes |  |
| Kutan Fenergioglu, 2009b | Turkey | Unclear | 159 | | SR | Length, HC | Fair | Yes |  |
| Bolat, 2012 | Turkey | Prospective cohort | 1,175 | | SR | Length, HC | Good | Yes |  |
| Bosley, 1981 | UK | Unclear | 320 | | SR | Length, HC | Good | Yes |  |
| D'Souza, 1981 | UK | Prospective cohort | 452 | | SR | Length, HC | Fair | No |  |
| Scott, 1981 | UK | Unclear | 855 | | SR | SGA | Good | No |  |
| Ounsted, 1985 | UK | Retrospective cohort | 1,392 | | SR | SGA | Good | No |  |
| Haste, 1991 | UK | Cross-sectional | 1,513 | | SR | Length, HC | Fair | No |  |
| Ong, 2002 | UK | Unclear | 1,335 | | SR | Length, HC | Poor | Yes |  |
| Pringle, 2005 | UK | Prospective cohort | 1,215 | | SR | Length, HC | Fair | Yes |  |
| Hindmarch, 2008 | UK | Unclear | 1,218 | | SR | HC | Good | No |  |
| Delpisheh, 2008 | UK | Case-control | 270 | | SR + biomarker | SGA, Length | Poor | Yes |  |
| Delpisheh, 2009 | UK | Case-control | 270 | | SR + biomarker | Length, HC | Fair | Yes |  |
| Prabhu, 2010 | UK | Prospective cohort | 1,924 | | SR | Length | Good | No |  |
| Lynch, 2011 | UK | Case-control | 67 | | SR + biomarker | SGA | Fair | No |  |
|  | **North America** |  |  | |  |  |  |  |  |
| Chernick, 1983 | Canada | Case-control | 70 | | SR | Length, HC | Good | Yes |  |
| Fried, 1987 | Canada | Prospective cohort | 667 | | SR | Length, HC | Fair | No |  |
| Beaulac-Baillargeon, 1987 | Canada | Unclear | 913 | | SR | Length, HC | Good | No |  |
| Arbuckle, 1989 | Canada | Prospective cohort | 806 | | SR | SGA | Good | No |  |
| Godel, 1992 | Canada | Cross-sectional | 162 | | SR | Length, HC | Good | Yes |  |
| Muscati, 1994 | Canada | Prospective cohort | 1,330 | | SR | SGA, Length, HC | Fair | Yes |  |
| Muscati, 1996 | Canada | Retrospective cohort | 1,339 | | SR | SGA | Good | No |  |
| Eliopoulos, 1996 | Canada | Prospective cohort | 94 | | SR + biomarker | Length, HC | Good | Yes |  |
| Perkins, 1997 | Canada | Unclear | 3,220 | | SR + biomarker | SGA | Fair | No |  |
| Millar, 1998 | Canada | Retrospective cohort | 4,181 | | SR | SGA | Fair | Yes |  |
| Fried, 1999 | Canada | Prospective cohort | 190 | | SR | Length, HC | Good | Yes |  |
| Infante-Rivard, 2006 | Canada | Unclear | 965 | | SR | SGA | Good | Yes |  |
| Infante-Rivard, 2007 | Canada | Case-control | 965 | | SR | SGA | Good | No |  |
| Mehaffey, 2010 | Canada | Cross-sectional | 918 | | SR | SGA | Fair | No |  |
| Almeida, 2011 | Canada | Case-control | 431 | | SR + biomarker | SGA | Fair | Yes |  |
| Kahn, 2011 | Canada | Prospective cohort | 5,337 | | SR + biomarker | SGA | Fair | No |  |
| Campbell, 2012 | Canada | Prospective cohort | 2,195 | | SR | SGA | Good | Yes |  |
| Erickson, 2012 | Canada | Retrospective cohort | 237,470 | | SR | SGA | Fair | Yes |  |
| Heaman, 2013 | Canada | Retrospective cohort | 6,421 | | SR | SGA | Good | Yes |  |
| Sánchez-Zamorano, 2004 | Mexico | Prospective cohort | 793 | | SR | Length | Poor | No |  |
| Luke, 1981 | USA | Cross-sectional | 637 | | SR | Length, HC | Fair | Yes |  |
| Naeye, 1981 | USA | Unclear | 8,193 | | SR | Length, HC | Fair | Yes |  |
| Harrison, 1983 | USA | Prospective cohort | 285 | | SR | Length, HC | Fair | No |  |
| Scholl, 1986 | USA | Prospective cohort | 775 | | SR | SGA | Fair | Yes |  |
| Wertelecki, 1987 | USA | Case-control | 925 | | SR | Length | Fair | Yes |  |
| Tenovuo, 1988 | USA | Case-control | 118 | | SR | SGA | Fair | No |  |
| Miller, 1989 | USA | Prospective cohort | 1,757 | | SR | Length | Poor | No |  |
| Callan, 1990 | USA | Case-control | 3,208 | | SR | SGA | Fair | No |  |
| Wen, 1990 | USA | Cross-sectional | 17,149 | | SR | SGA | Good | No |  |
| Fox, 1990 | USA | Prospective cohort | 714 | | SR + biomarker | Length | Good | No |  |
| Wen, 1990 | USA | Prospective cohort | 15,539 | | SR | SGA | Fair | Yes |  |
| Day, 1992 | USA | Prospective cohort | 763 | | SR | Length, HC | Fair | No |  |
| Lang, 1992 | USA | Case-control | 772 | | SR | SGA | Fair | Yes |  |
| Castro, 1993 | USA | Retrospective cohort | 7,741 | | SR | SGA | Fair | Yes |  |
| Goldenberg, 1993 | USA | Cross-sectional | 1,205 | | SR | Length, HC | Poor | No |  |
| Jacobson, 1994a | USA | Cross-sectional | 412 | | SR | Length, HC | Good | No |  |
| Jacobson, 1994b | USA | Cross-sectional | 417 | | SR | Length, HC | Fair | No |  |
| Cornelius, 1995 | USA | Prospective cohort | 310 | | SR | SGA, Length, HC | Fair | No |  |
| Cliver, 1995 | USA | Prospective cohort | 1,205 | | SR | Length, HC | Fair | No |  |
| Zhang, 1995 | USA | Unclear | 3,861 | | SR | SGA | Good | No |  |
| Lang, 1996 | USA | Cross-sectional | 9,490 | | SR | SGA | Fair | Yes |  |
| Wang, 1997 | USA | Prospective cohort | 740 | | SR + biomarker | Length, HC | Fair | Yes |  |
| Schramm, 1997 | USA | Prospective cohort | 176,843 | | SR | SGA | Good | No |  |
| Lindsay, 1997 | USA | Prospective cohort | 129 | | SR | Length, HC | Fair | Yes |  |
| Wu, 1998 | USA | Retrospective cohort | 9,402 | | SR | SGA | Good | Yes |  |
| Sprauve, 1999 | USA | Case-control | 945 | | SR | SGA | Fair | No |  |
| Bernstein, 2000 | USA | Prospective cohort | 101 | | SR + biomarker | HC | Fair | No |  |
| Shankaran, 2004 | USA | Case-control | 410 | | SR | Length, HC | Poor | Yes |  |
| Salihu, 2005 | USA | Retrospective cohort | 327,802 | | SR + biomarker | SGA | Fair | Yes |  |
| Schwendemann, 2005 | USA | Prospective cohort | 11,827 | | SR | SGA | Fair | Yes |  |
| Salihu, 2005 | USA | Retrospective cohort | 7,792,990 | | SR + biomarker | SGA | Fair | Yes |  |
| Fitzgerald, 2007 | USA | Retrospective cohort | 266,782 | | SR | SGA | Good | Yes |  |
| Okah, 2007 | USA | Retrospective cohort | 5,107 | | SR | SGA | Fair | Yes |  |
| England, 2007 | USA | Cross-sectional | 4,289 | | SR + biomarker | SGA | Fair | Yes |  |
| Aagaard-Tillery, 2008 | USA | Cross-sectional | 424,912 | | SR | SGA | Fair | Yes |  |
| Polakowski, 2009 | USA | Retrospective cohort | 915,441 | | SR | SGA | Fair | Yes |  |
| Leviton, 2010 | USA | Prospective cohort | 1,004 | | SR | HC | Good | No |  |
| Wen, 2010 | USA | Retrospective cohort | 1,370 | | SR + biomarker | SGA | Good | No |  |
| Gray, 2010 | USA | Cross-sectional | 87 | | SR + biomarker | Length, HC | Fair | Yes |  |
| Okah, 2010 | USA | Retrospective cohort | 11,864 | | SR | SGA | Good | Yes |  |
| Aagaard-Tillery, 2010 | USA | Prospective cohort | 5,188 | | SR | SGA | Fair | Yes |  |
| Espy, 2011 | USA | Prospective cohort | 304 | | SR + biomarker | Length, HC | Fair | Yes |  |
| England, 2012* | USA | Retrospective cohort | 502 | | SR | Length, HC | Fair | No |  |
| Quesada, 2012 | USA | Unclear | 2,808 | | SR | SGA | Poor | Yes |  |
| Goetzinger, 2012 | USA | Retrospective cohort | 65,104 | | SR | SGA | Good | No |  |
| Himes, 2013 | USA | Prospective cohort | 119 | | SR + biomarker | Length, HC | Fair | Yes |  |
| Hinkle, 2014 | USA | Retrospective cohort | 25,241 | | SR + biomarker | SGA | Fair | No |  |
| Xaverius, 2014 | USA | Retrospective cohort | 141,579 | | SR | SGA | Fair | Yes |  |
| Seravalli, 2014 | USA | Prospective cohort | 1,982 | | SR | SGA | Good | No |  |
| Harrod, 2015  Blatt, 2015 | USA  USA | Prospective cohort  Retrospective cohort | 670  927,424 | | SR  SR | SGA, Length, HC  SGA | Fair  Good | Yes  Yes |  |
| Ahmadi-Montecalvo, 2016 | USA | Cross-sectional | 886 | | SR | SGA | Fair | Yes |  |
| Xie, 2016 | USA | Prospective cohort | 1,100 | | SR | SGA, Length | Fair | Yes |  |
|  | **Oceania** |  |  | |  |  |  |  |  |
| Newnham, 1990 | Australia | Prospective cohort | 535 | | SR | HC | Fair | No |  |
| Read, 1993 | Australia | Case-control | 1,664 | | SR | SGA | Fair | Yes |  |
| O'Callaghan, 1997 | Australia | Case-control | 165 | | SR | SGA | Fair | No |  |
| Kleijer, 2005 | Australia | Case-control | 788 | | SR | SGA | Fair | No |  |
| Panaretto, 2006 | Australia | Prospective cohort | 456 | | SR | SGA | Good | Yes |  |
| Burns, 2008 | Australia | Cross-sectional | 407,957 | | SR | SGA | Fair | Yes |  |
| Quinton, 2008 | Australia | Case-control | 41 | | SR | HC | Good | No |  |
| Bickerstaff, 2012 | Australia | Retrospective cohort | 30,524 | | SR | SGA | Poor | Yes |  |
| Hodyl, 2014a  Hodyl, 2014b | Australia  Australia | Retrospective cohort  Retrospective cohort | 171,991  172,305 | | SR  SR | SGA  SGA | Fair  Fair | Yes  Yes |  |
| Thompson, 2001 | New Zealand | Case-control | 1,714 | | SR | SGA | Fair | Yes |  |
| Gao, 2006 | New Zealand | Prospective cohort | 1,324 | | SR | SGA | Fair | Yes |  |
| Mitchel, 2008 | New Zealand | Case-control | 1,714 | | SR | SGA | Good | Yes |  |
| Anderson, 2013 | New Zealand | Retrospective cohort | 26,159 | | SR | SGA | Poor | Yes |  |
| McCowan, 2016 | New Zealand | Prospective cohort | 5,606 | | SR | SGA | Fair | No |  |
| McCowan, 2009 | New Zealand, Australia | Prospective cohort | 2,504 | | SR | SGA | Fair | No |  |
|  | **South America** |  |  | |  |  |  |  |  |
| Barros, 1992 | Brazil | Prospective cohort | 5,914 | | SR | SGA | Fair | Yes |  |
| Horta, 1997 | Brazil | Retrospective cohort | 5,166 | | SR | SGA | Good | Yes |  |
| Zambonato, 2004 | Brazil | Cross-sectional | 911 | | SR | SGA | Fair | Yes |  |
| Gonçalves-Silva, 2005 | Brazil | Cross-sectional | 2,037 | | SR | Length | Good | No |  |
| Galão, 2009 | Brazil | Cross-sectional | 718 | | SR | SGA | Fair | No |  |
| Gonçalves-Silva, 2009 | Brazil | Cross-sectional | 1,437 | | SR | Length | Good | No |  |
| Zhang, 2011 | Brazil | Cross-sectional | 2,557 | | SR | Length | Fair | Yes |  |
| Matijasevich, 2011 | Brazil | Prospective cohort | 9,591 | | SR | Length, HC | Good | No |  |
| Nunes, 2015 | Brazil | Cross-sectional | 241 | | SR | HC | Fair | No |  |
| Berlanga, 2002 | Chile | Prospective cohort | 46 | | SR + biomarker | Length | Fair | Yes |  |
| Gray, 2008 | Uruguay | Cross-sectional | 49 | | SR + biomarker | Length, HC | Fair | Yes |  |
|  | **Multiple** |  |  | |  |  |  |  |  |
| Zeitlin, 2001 | Multi-country ^a^ | Case-control | 11,160 | | SR | SGA, Length | Fair | No |  |
| Victora, 2015 | Multi-country ^b^ | Cross-sectional | 51,200 | | SR | Stunting | Good | No |  |
| McCowan, 2010 | Multi-country ^c^ | Prospective cohort | 3,513 | | SR | SGA | Good | Yes |  |

Multi-country studies: ^a^, Czech Republic, Finland, France, Germany, Greece, Hungary, Ireland, Italy, The Netherlands, Poland, Romania, Russia, Scotland (UK), Slovenia, Spain, Sweden; ^b^, Brazil, China, India, Italy, Kenya, Oman, England, USA; ^c^, New Zealand, Australia, UK, Ireland. Abbreviations: SR, self-reported; SGA, small for gestational age; HC, head circumference.

* These studies include use of smokeless tobacco (snuff); all other studies refer to smoked tobacco only (cigarettes).
