# Supplementary material for: The association between active tobacco use during pregnancy and growth outcomes of children under five years of age: a systematic review and meta-analysis
Source: BMC Public Health. 2018 Dec 13;18:1372. doi: 10.1186/s12889-018-6137-7 (PMC6293508; doi:10.1186/s12889-018-6137-7)
Supplement: Supplementary file 4 — Table S2. Inclusion and exclusion criteria for selecting studies for the systematic review and meta-analysis. Description of data: A complete list of inclusion and exclusion criteria used to select the studies for the systematic review and meta-analysis. (DOCX 19 kb) [file 12889_2018_6137_MOESM4_ESM.docx]

**Additional file 4: Table S2**. Inclusion and exclusion criteria for selecting studies for the systematic review and meta-analysis.

| Inclusion criteria | Exclusion criteria |
| --- | --- |
| Systematic review   - Published articles - Publication period: Jan 1, 1980 – Oct 31, 2016 - English, French, Portuguese, Spanish - Any study design with individual-level data - Active tobacco use during pregnancy - Outcome: SGA (<10^th^ percentile or >2 SD of standard or reference), length/height, stunting, head circumference - Children < 5 years of age - Term births (≥37 weeks of gestation) - Analysis of association between exposure and outcomes   Meta-analysis   - Comparable exposure and outcome measures - Adjusted OR - Confidence intervals given for OR - Standard deviation and N given for MD | - Conference abstracts, grey literature - Published outside of this period - Other languages - Ecological studies (population-level data) - Passive exposure or active tobacco use before or after pregnancy only - Outcome: SGA (or IUGR, FGR) with different definitions, low birth weight only, head diameter only - Children >5 years of age - Preterm births (<37 weeks of gestation) - Descriptive analyses - Incomparable exposure and outcome measures - Crude OR - Confidence intervals not given for OR - Standard deviation and N not given for MD |
