# Supplementary material for: The association between active tobacco use during pregnancy and growth outcomes of children under five years of age: a systematic review and meta-analysis
Source: BMC Public Health. 2018 Dec 13;18:1372. doi: 10.1186/s12889-018-6137-7 (PMC6293508; doi:10.1186/s12889-018-6137-7)
Supplement: Supplementary file 5 — Table S3. Quality assessment criteria. Description of data: Twelve quality assessment criteria standards and the number of points assigned to each. (DOCX 18 kb) [file 12889_2018_6137_MOESM5_ESM.docx]

**Additional file 5: Table S3**. Quality assessment criteria.

| **Quality assessment criteria** | **Points** |
| --- | --- |
| 1. Was the research question or objective clearly stated? 2. Was the study population clearly specified and defined? 3. Were there pre-specified inclusion and/or exclusion criteria? 4. Was the exposure clearly defined and valid? 5. Was the outcome clearly defined and valid? 6. Was a biomarker used to assess exposure? 7. Did the study examine different levels of exposure? 8. Was the exposure assessed more than once over time? 9. Was the outcome assessed more than once over time? 10. Were outcome assessors blinded to the exposure status? 11. Were potential confounders measured and statistically adjusted for? 12. For case-control studies, were controls selected from the same or similar   population that gave rise to the cases, and in the same timeframe? | 3 points  2 points  1 point  3 points  3 points  3 points  2 points  2 points  1 points  1 point  3 points  2 points |
