# Supplementary figures and images for: The association between active tobacco use during pregnancy and growth outcomes of children under five years of age: a systematic review and meta-analysis
Source: BMC Public Health. 2018 Dec 13;18:1372. doi: 10.1186/s12889-018-6137-7 (PMC6293508; doi:10.1186/s12889-018-6137-7)

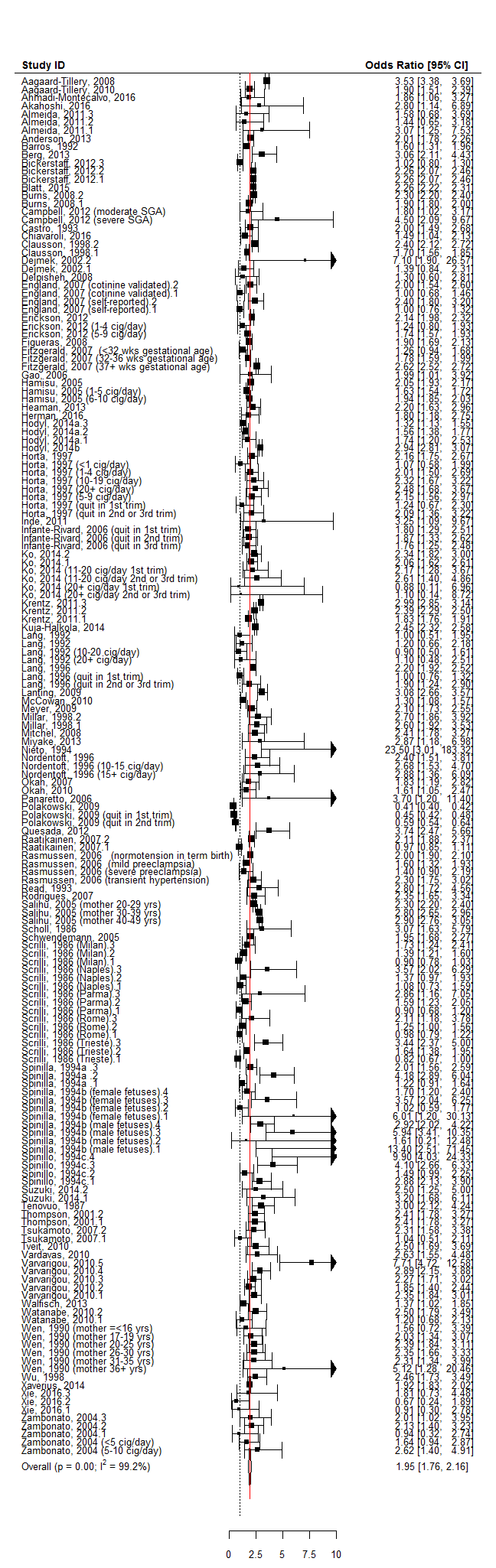

Supplement: Supplementary file 7 — Figure S1. Effect of all tobacco use during pregnancy on SGA at birth. Description of data: A forest plot illustrating the effect of all types of tobacco exposure during pregnancy on SGA at birth as indicated by the Odds Ratio and 95% CI. (PNG 73 kb) [file 12889_2018_6137_MOESM7_ESM.png]

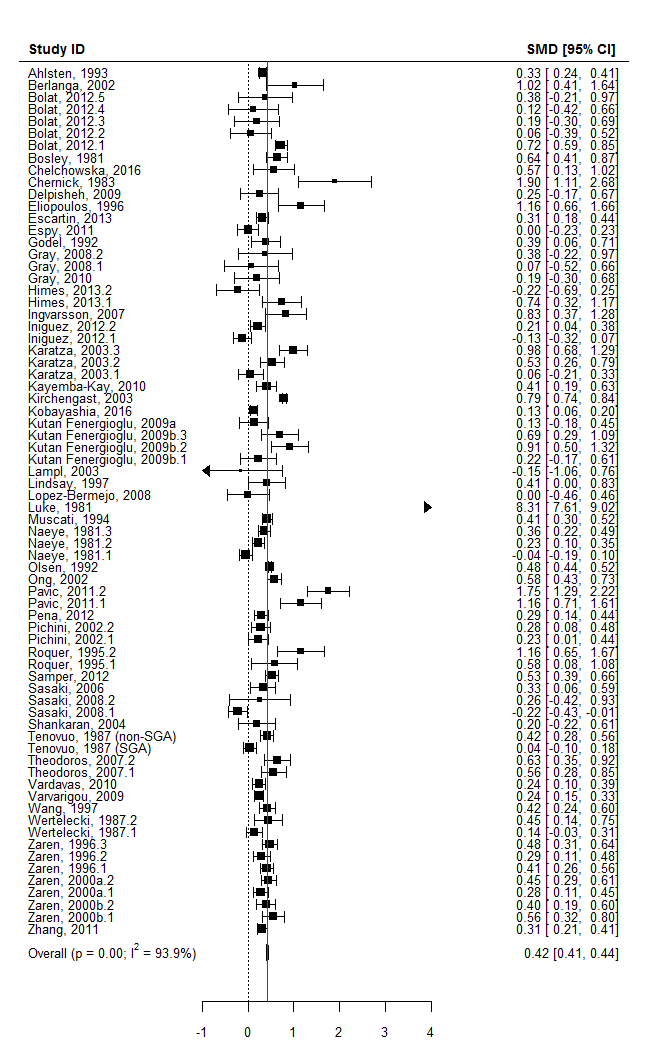

Supplement: Supplementary file 8 — Figure S2. Effect of all tobacco use during pregnancy on length at birth. Description of data: A forest plot illustrating the effect all types of tobacco use and exposure during pregnancy on length at birth as indicated by standardized mean differences (95% CI). (PNG 30 kb) [file 12889_2018_6137_MOESM8_ESM.png]

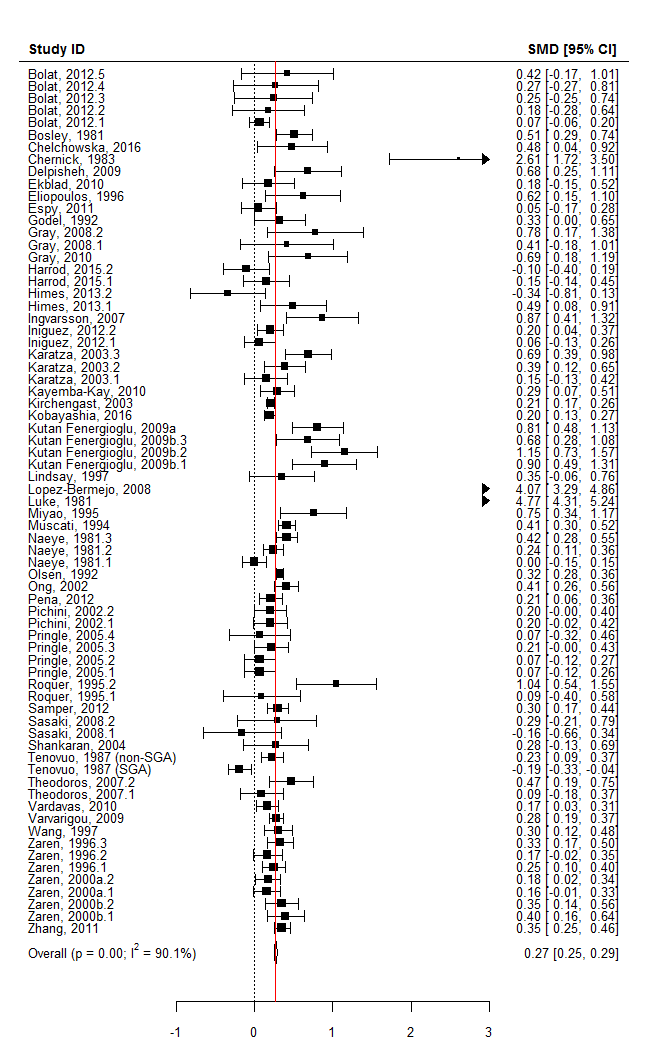

Supplement: Supplementary file 9 — Figure S3. Effect of all tobacco use during pregnancy on head circumference at birth. Description of data: A forest plot illustrating the effect of all tobacco use during pregnancy on head circumference at birth as indicated by standardized mean differences (95% CI). (PNG 29 kb) [file 12889_2018_6137_MOESM9_ESM.png]
